# A Novel Gear Fault Diagnosis Method Based on COA-VMD, RCMFE and SSA-BP

**DOI:** 10.3390/e28101121

**Published:** 2026-10-14

**Authors:** Duowen Chen, Haoze Lv, Xinze Li, Wenjie Liang, Kailin Bai, Xin Zhao, Linlin Cao, Xiaolong Zhou

**Affiliations:** Mechanical Engineering College, Beihua University, Jilin 132021, China; chinachenduowen@gmail.com (D.C.);

**Keywords:** fault diagnosis, coati optimization algorithm, variational mode decomposition, refined composite multiscale fuzzy entropy, sparrow search algorithm, BP neural network

## Abstract

Aiming at the difficulties in extracting gear fault feature information and the unsatisfactory performance of commonly used machine learning classification models in terms of feature separability and stable within-distribution recognition, this paper proposes a fault diagnosis method combining variational mode decomposition (VMD) optimized by the coati optimization algorithm (COA) and a BP neural network optimized by the sparrow search algorithm (SSA). Firstly, a composite index based on permutation entropy and mutual information entropy is adopted as the fitness function, and the COA is used to optimize the key parameters of VMD to improve the decomposition accuracy of the VMD algorithm. Secondly, the intrinsic mode function (IMF) components sensitive to fault information are selected for signal reconstruction according to the time–frequency-domain comprehensive evaluation factor algorithm, and the refined composite multiscale fuzzy entropy (RCMFE) is used to extract the features of the reconstructed signal to form a state feature set. Finally, the SSA is employed to optimize the parameters of the BP model to achieve accurate diagnosis of gear working conditions and fault types. The proposed method is verified using measured gear single and compound fault data and compared with commonly used signal decomposition methods and machine learning classification models. The results show that the proposed method can effectively extract gear fault feature information and has the advantages of high accuracy, good feature separability and stable within-distribution recognition. It can achieve ideal diagnosis accuracy for both single and compound gear faults, providing a certain reference for solving such problems.

## 1. Introduction

Gears, as critical components of rotating machinery, occupy a pivotal position in industry due to their efficient and adjustable transmission characteristics [1]. However, with the development of large-scale and complex industrial equipment, gears often operate under harsh environments such as high temperature, high pressure and strong corrosion, making them prone to failures such as wear, tooth crack and tooth breakage, which further degrade equipment performance and threaten operational safety [2]. Therefore, research on gear fault diagnosis methods is of great engineering significance.

Gear vibration signals collected by sensors usually contain fault shock, background information and environmental noise, exhibiting strong nonlinearity, non-stationarity and volatility, which pose significant challenges to signal processing and fault identification [3].

To improve the accuracy of fault identification, researchers have proposed various signal processing methods, such as empirical mode decomposition (EMD) and ensemble empirical mode decomposition (EEMD) [4,5]. He et al. [6] used EMD to diagnose slight eccentricity faults of gears in circular knitting machines. Tao et al. [7] achieved a recognition rate of 94.17% for planetary gear signals processed by EEMD, obtaining relatively ideal results. Zhang et al. [8] effectively extracted gearbox fault features based on the EEMD signal decomposition method and realized accurate fault diagnosis. Nevertheless, these methods suffer from inherent drawbacks such as endpoint effect, mode aliasing and noise residue [9,10]. To overcome these limitations, variational mode decomposition (VMD) [11] was proposed and has been widely applied in gear fault diagnosis with satisfactory results. Wang et al. [12] proposed a VMD-based gear fault feature extraction method for scraper conveyor gearboxes, which can effectively eliminate redundant signals and improve signal purity. Lin et al. [13] combined VMD with probabilistic neural networks to effectively solve the problems of mode aliasing and poor adaptability common in recursive signal processing methods, achieving effective gear fault diagnosis. Cao et al. [14] proposed a wind turbine gearbox fault diagnosis approach combining sparrow search algorithm optimized variational mode decomposition and improved refined composite multi-scale dispersion entropy. They performed signal decomposition and denoising via SSA-optimized VMD with correlation coefficient-based reconstruction, extracted fault features using the improved entropy metric, and achieved accurate gear fault classification with SVM after GPLVM-based dimensionality reduction.

In addition to signal-processing-based approaches, deep learning and multi-source information fusion have been increasingly adopted. Yang et al. [15] proposed a hybrid deep model fusing displacement error and pressure signals for hydraulic cylinder fault diagnosis under complex conditions, and Wang et al. [16] developed an improved multi-target domain adversarial network to enhance the antinoise ability and robustness of transfer diagnosis for rotating machinery. Apart from vibration analysis, visual monitoring provides another effective means; for example, Zuo et al. [17] established an end-to-end bolt key-point detection model for industrial magnetic separator systems under varying illumination and stains.

Although VMD-based gear fault diagnosis technologies have achieved certain results, some issues still need to be addressed. The decomposition performance of VMD heavily depends on the settings of two key parameters: the number of modal components K and the quadratic penalty factor α. Traditional manual parameter tuning methods are computationally intensive and may ignore the interaction between parameters. Intelligent optimization algorithms, such as grey wolf optimization (GWO) [18] and the whale optimization algorithm (WOA) [19], have been widely used to optimize VMD parameters. The coati optimization algorithm (COA) [20] is a novel intelligent optimization algorithm with stronger global optimization capability and higher search efficiency. Applying the COA to VMD parameter optimization will further enhance VMD performance and ensure the accuracy of signal decomposition.

Meanwhile, identifying how to extract effective fault features from gear vibration signals remains one of the core difficulties in gear fault diagnosis. In recent years, to effectively extract fault features and quantify the complexity of vibration signals, researchers have developed nonlinear dynamic feature extraction methods such as permutation entropy (PE), sample entropy (SE) and dispersion entropy (DE), which have been widely used in fault diagnosis research [11,12,13,14,15,16,17,18,19,20,21,22,23]. However, these methods only evaluate signal complexity on a single time scale, resulting in limited feature information and sometimes failing to achieve ideal recognition results. To address this issue, researchers proposed the multiscale entropy (MSE) algorithm [24]. Nevertheless, when dealing with long data sequences, these methods may suffer from slow computation speed, severe loss of fault information and unclear influence of amplitude relationships between signals. Refined composite multiscale fuzzy entropy (RCMFE) improves calculation accuracy by further refining the sequences corresponding to each scale factor [25]. This makes RCMFE exhibit higher sensitivity and accuracy in feature extraction, which helps improve the performance of fault diagnosis models.

In terms of fault classification, since the number of collected samples is often very limited, machine learning methods suitable for small samples such as k-nearest neighbor (KNN) and decision tree (DT) are widely used. However, the performance of these methods is easily affected by parameters, leading to unstable diagnosis results. The sparrow search algorithm (SSA) optimized back-propagation (BP) model uses the SSA to automatically determine the parameters of the BP model, which can effectively improve the applicability, stability and generalization ability of the network structure.

**Figure 1:**
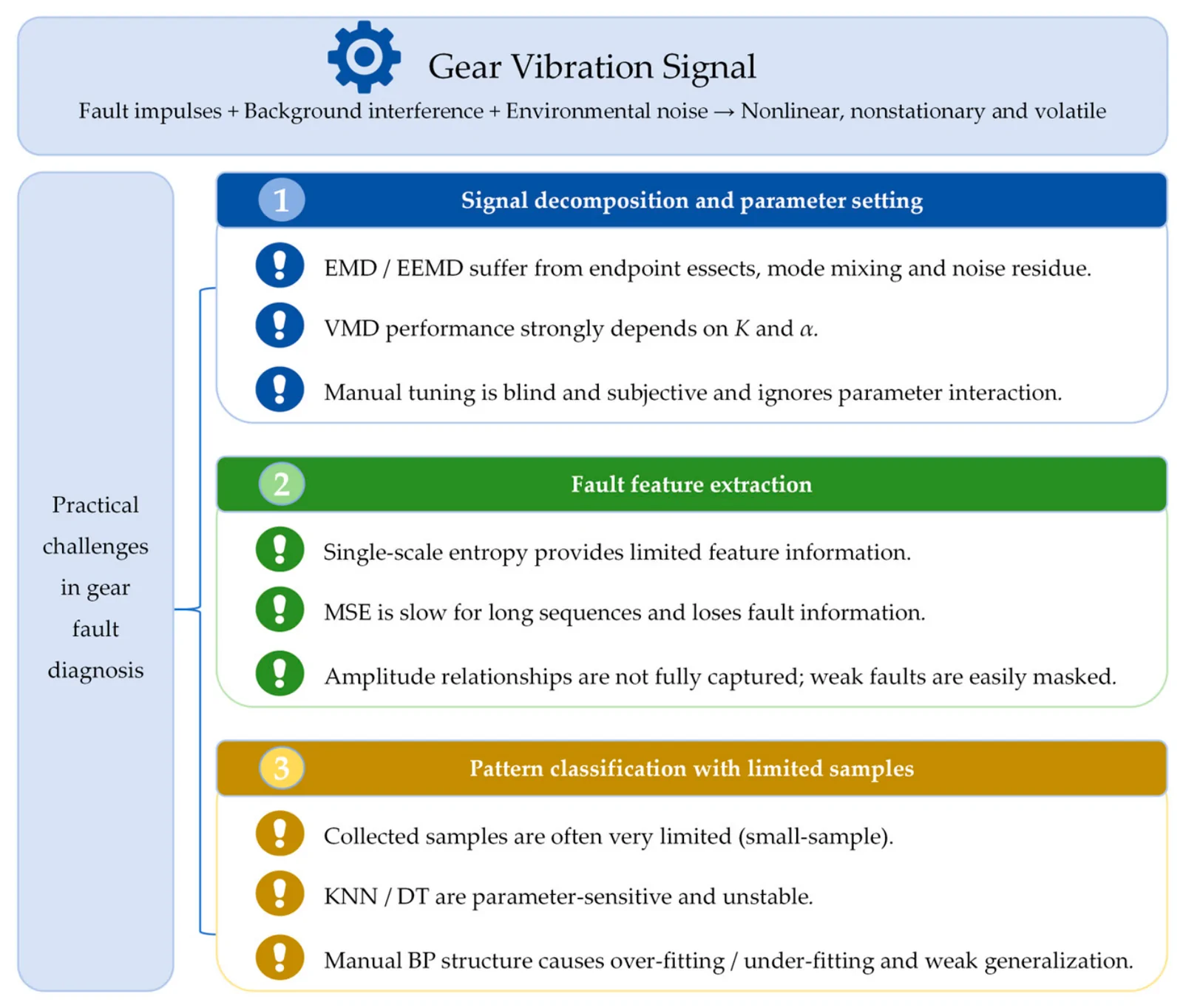
Practical challenges in gear fault diagnosis.

The practical challenges of gear fault diagnosis are summarized in Figure 1.

Based on the above analysis, this paper proposes a gear fault diagnosis method based on COA-VMD, RCMFE and SSA-BP. The main contributions of this paper are summarized as follows:

(1) To eliminate the subjectivity of manual tuning of the two key VMD parameters *K* and *α*, a composite index (CI) that integrates permutation entropy and mutual information entropy is constructed as the fitness function, and the COA, an existing swarm intelligence algorithm, is adopted to optimize *K* and *α* simultaneously, thereby suppressing mode aliasing and improving decomposition accuracy.
(2) To avoid the loss of key information caused by using a single time-domain or frequency-domain criterion when discarding false IMF components, a new time–frequency-domain comprehensive evaluation factor (CEF), which fuses the energy entropy increment with the frequency–domain cross-correlation coefficient, is proposed to automatically select fault-sensitive IMFs for reconstruction so that the reconstructed signal retains more fault information.
(3) To address the unstable recognition rate and weak generalization of common machine learning models with limited samples, the sparrow search algorithm (SSA), an existing optimizer, is used to determine the optimal number of hidden layer neurons of a two-layer BP neural network, ensuring an appropriate model structure for accurate fault identification. Experiments on measured single and compound gear faults, together with comparisons with common decomposition and classification methods, show that the proposed method achieves ideal diagnostic accuracy for both single and compound faults, providing a new solution for gear fault diagnosis.

The rest of this paper is organized as follows. Section 2 provides a brief overview of the fundamental theories. Section 3 describes the diagnostic process of the proposed method in detail. Section 4 introduces the experimental design. Section 5 discusses the application of this method in gear single fault and compound fault diagnosis. Section 6 presents the main conclusions and future research directions.

## 2. Fundamental Theories

### 2.1. VMD Algorithm

The core idea of the VMD algorithm is to convert a signal into a variational problem model and then search for the optimal solution of the model through iterative updates. Specifically, it continuously iterates the center frequency and bandwidth of each modal component to realize adaptive decomposition of each frequency band of the signal and decomposes the original signal into a series of IMF components with different center frequencies and bandwidths. The detailed theoretical derivation of VMD can be found in reference [11].

### 2.2. Determination of Key Parameters of VMD Based on the COA

The COA is a recently proposed swarm intelligence optimization algorithm inspired by the collective behaviors of coati populations in nature. Its core mechanism is built upon the mathematical modeling of two distinctive natural behaviors of coatis: (i) cooperative hunting and attacking of iguanas and (ii) escape strategies from predators. These two behaviors correspond to the global exploration and local exploitation capabilities of the algorithm, respectively, enabling the COA to effectively balance search breadth and solution precision in complex optimization spaces.

#### 2.2.1. Cooperative Hunting and Attacking Strategy

The first phase of the COA simulates the cooperative hunting behavior of coatis preying on iguanas. In this strategy, the coati population is evenly divided into two subgroups. The first subgroup *β* climbs trees to locate and scare iguanas, forcing them to fall to the ground. The second subgroup *δ* waits on the ground to ambush and capture the fallen iguanas. This distributed cooperative search mechanism endows the COA with strong global exploration ability, allowing it to thoroughly explore the entire solution space and avoid premature convergence to local optima.

In the algorithmic implementation, the position of the best individual in the current population is designated as the position of the iguana (the optimal food source). The position update rules for the two subgroups of coatis are mathematically formulated as follows:

For the coatis in the first subgroup *β* climbing trees, their positions are updated according to:

(1) $$x_{i,j}^{P1}=x_{i,j}+r\cdot \left({\mathrm{Iguana}}_{j}-I\cdot x_{i,j}\right),\;i=1,2,\ldots ,\lfloor N/2\rfloor ,\;j=1,2,\ldots ,m$$

After the iguana falls to the ground, it is randomly repositioned within the search space using:

(2) $${\mathrm{Iguana}}_{j}^{G}=lb_{j}+r\cdot (ub_{j}-lb_{j})$$

For the coatis in the second subgroup *δ* waiting on the ground, their positions are updated based on the new position of the iguana:

(3) $$\begin{array}{l} x_{i,j}^{P1}=\left\{\begin{array}{l} x_{i,j}+r\cdot ({\mathrm{Iguana}}_{j}^{G}-I\cdot x_{i,j})\;\;\;\;F_{{\mathrm{Iguana}}^{G}}<F_{i}\\ x_{i,j}+r\cdot (x_{i,j}-{\mathrm{Iguana}}_{j}^{G})\;\;\;\;\;\;\;\;\;F_{{\mathrm{Iguana}}^{G}}\geq F_{i} \end{array}\right.\\ i=\left\lfloor N/2\right\rfloor +1,\left\lfloor N/2\right\rfloor +2,\ldots ,N.\;j=1,2,\ldots ,m. \end{array}$$

A greedy selection mechanism is employed to determine whether the newly calculated position is accepted. A coati will update its position only if the new position yields a better objective function value; otherwise, it remains at its original position:

(4) $$X_{i}=\left\{\begin{array}{l} X_{i}^{P1}\;\;\;\;\;F_{i}^{P1}<F_{i}\\ X_{i}\;\;\;\;\;\;\;F_{i}^{P1}\geq F_{i}\; \end{array}\right.$$

where *N* is the population size, *m* is the dimensionality of the optimization problem, *x_i,j_* is the *j*-th dimension of the *i*-th coati at the current iteration, r is a random number uniformly distributed in [0,1], *Iguana_j_* is the *j*-th dimension of the current best individual’s position, *I* is an integer randomly selected from the set {1,2}, ${\mathrm{Iguana}}_{j}^{G}$ is the *j*-th dimension of the randomly repositioned iguana, *lb_j_* and *ub_j_* are the lower and upper bounds of the *j*-th decision variable, respectively, *F_i_* is the objective function value of the *i*-th coati, and ⌊⋅⌋ denotes the floor function.

#### 2.2.2. Predator Escape Strategy

The second phase of the COA models the escape behavior of coatis when encountering predators. When a predator is detected, coatis do not flee to distant areas but instead move to safe positions in the immediate vicinity of their current location. This behavior enables the COA to perform intensive local search around promising solutions, thereby improving the convergence accuracy of the algorithm.

To simulate this local escape behavior, the search range is dynamically reduced as the iteration progresses. The local search bounds for the *j*-th dimension at iteration *t* are defined as:

(5) $$lb_{j}^{\mathrm{local}}=\frac{lb_{j}}{t},\;ub_{j}^{\mathrm{local}}=\frac{ub_{j}}{t}\;t=1,2,\ldots ,T$$

where *t* is the current iteration number and *T* is the maximum number of iterations.

The position update rule for coatis during the escape phase is given by:

(6) $$x_{i,j}^{P2}=x_{i,j}+(1-2r)\cdot \left(lb_{j}^{\mathrm{local}}+r\cdot \left(ub_{j}^{\mathrm{local}}-lb_{j}^{\mathrm{local}}\right)\right)$$

Similar to the hunting phase, a greedy selection strategy is applied to ensure that only superior positions are retained:

(7) $$X_{i}=\left\{\begin{array}{l} X_{i}^{P2}\;\;\;\;\;F_{i}^{P2}<F_{i}\\ X_{i}\;\;\;\;\;\;\;\;F_{i}^{P2}\geq F_{i}\;\; \end{array}\right.$$

where $x_{i,j}^{P2}$ is the *j*-th dimension of the *i*-th coati’s new position calculated in the second phase and $F_{i}^{P2}$ is the corresponding objective function value.

The COA iteratively executes the above two phases until the predefined termination condition (maximum number of iterations or convergence threshold) is satisfied. The individual with the best objective function value at the end of the iteration process is output as the optimal solution to the problem.

#### 2.2.3. Determination of the Fitness Function

The key to optimizing VMD parameters using artificial intelligence search algorithms lies in defining an appropriate fitness function. This function can quantify the performance of the VMD algorithm under the current parameter configuration and update the parameters based on the quantification results to optimize the overall performance [26].

To address the limitations of single indices, which cannot effectively reflect signal characteristics [27] and exhibit poor adaptability to different operating conditions, this paper proposes a composite index combining permutation entropy (PE) and mutual information entropy (MIE). PE is a widely used method for detecting signal complexity, characterized by easy implementation and strong robustness, making it particularly suitable for analyzing nonlinear signals. When an IMF component contains significant noise and exhibits high complexity and randomness, its PE value is large; conversely, when the signal is highly regular, its PE value is small.

When calculating the PE value *H*(*u_i_*) of each IMF component, the calculation parameters of PE need to be predetermined. Typically, the embedding dimension *m* ranges from three to eight, and the delay time *τ* is set to 1. A larger value of m leads to higher computational complexity. To improve computational efficiency, *m* = 3 is adopted in this study. MIE measures the strength of correlation between two signals: a larger MIE value indicates a stronger correlation. For an IMF component, the richer the original signal feature information it contains, the larger the MIE value between the IMF component and the original signal.

The expression of the *CI* established in this paper is:

(8) $$\mathrm{CI}=\frac{H(u_{i})}{I(u_{i},u)}$$

where *H*(*u_i_*) is the permutation entropy value and *I*(*u_i_*,*u*) is the mutual information entropy value.

This composite index simultaneously considers both the noise level and feature information content of IMF components. A smaller CI value indicates that the corresponding IMF component contains less noise and richer feature information. Therefore, the minimum value of the composite index min {*CI*} is adopted as the fitness function for the optimization of key VMD parameters.

#### 2.2.4. The Steps to Optimize VMD with the COA

The steps to optimize VMD with the COA are as follows:

Step 1: Initialize the VMD parameter combination to be optimized and the core parameters of the COA.

Step 2: Calculate the fitness value of each coati individual in the population using the composite index fitness function defined in Section 2.2.3.

Step 3: Update the positions of tree-climbing coatis and ground-ambushing coatis in the cooperative hunting phase (global exploration phase) based on their fitness rankings.

Step 4: Update the positions of all coatis in the predator escape phase (local exploitation phase) according to their current fitness values.

Step 5: Map the updated position of each coati to the corresponding VMD parameter combination.

Step 6: Judge whether the predefined stopping condition (maximum number of iterations) is satisfied. If yes, output the globally optimal VMD parameter combination [*K*, *α*]; otherwise, return to Step 2 and continue the iterative optimization process until the stopping condition is met.

It should be clarified that the COA is an existing metaheuristic algorithm proposed by Dehghani et al. [20]. The present study does not claim to invent the COA itself; rather, its contribution lies in the problem-specific design of the PE-MIE composite fitness and the systematic integration for gear fault diagnosis.

**Figure 2:**
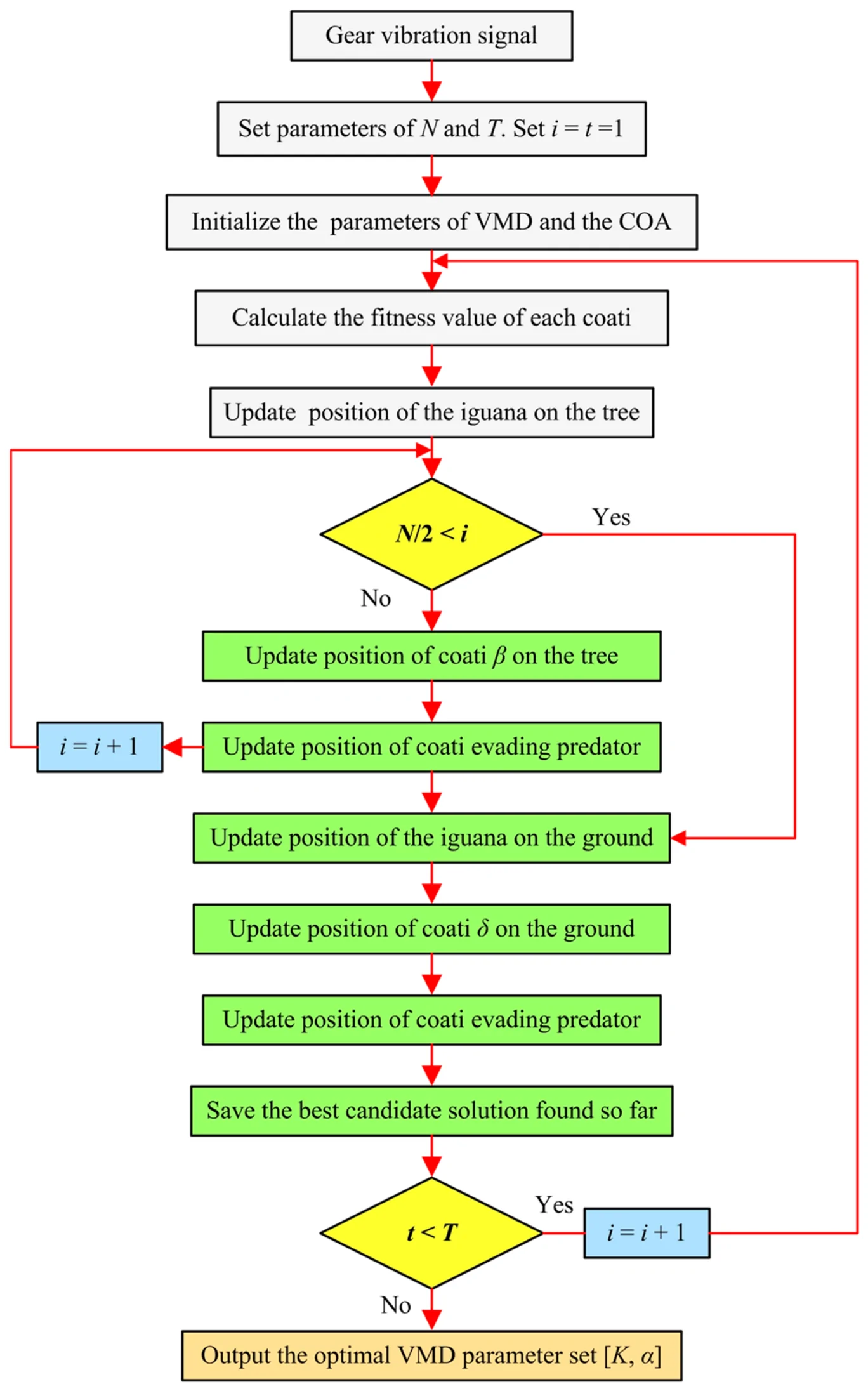
Flowchart of VMD optimization based on the COA.

A flowchart of the COA-based VMD key parameter optimization framework is illustrated in Figure 2.

### 2.3. Sensitive IMF Discrimination Algorithm Based on a Time–Frequency-Domain Comprehensive Evaluation Factor

Identifying how to obtain effective components from the IMF components decomposed by VMD is one of the decisive factors for the effectiveness of signal processing. Currently, the elimination and reconstruction of false IMFs based on a single time-domain or frequency-domain criterion cannot take into account both time-domain and frequency-domain characteristics of the signal, leading to the loss of key signal information and affecting the discrimination accuracy.

When a gear fault occurs, the meshing of faulty teeth will not only cause changes in the amplitude and phase of the vibration signal but also change the energy distribution of the vibration signal [28]. The IMF components obtained by VMD have center frequencies distributed from low to high and possess different energy levels. The main modal components characterizing the signal’s own features should occupy the dominant energy, while the false modal components insensitive to feature information account for a small proportion of energy [29]. The time-domain cross-correlation coefficient method is the most commonly used method for discriminating noise interference components in signals [30]. However, when noise exists in the signal, it will not only affect the signal’s own characteristics but also reduce the calculation accuracy of the time-domain cross-correlation coefficient. In contrast, the power spectral density of noise components in the frequency domain is small, and the power spectrum cross-correlation coefficient is less affected by noise [31]. Based on the above analysis, this paper proposes a sensitive IMF component discrimination method based on a time–frequency-domain CEF, which uses the energy entropy increment of each IMF component and the frequency-domain cross-correlation coefficient value to select sensitive modal components.

Suppose the gear vibration signal *x*(*t*) is decomposed by COA-VMD into *K* IMF components *u_i_*(*t*) (*i* = 1, 2, ∙∙∙, *K*). The specific calculation process of the sensitive IMF discrimination algorithm is as follows:

(1) Calculate the energy of each IMF component:
  (9) $$E(u_{i}(t))=\int_{0}^{T}u_{i}{\left(t\right)}^{2}\mathrm{dt}$$
(2) Calculate the normalized energy of each IMF component:
  (10) $$p(i)=\frac{E(u_{i}(t))}{\sum_{i=1}^{K}E(u_{i}(t))}$$
(3) Calculate the energy entropy increment value of each IMF component:
  Δ*q_i_* = −*p*(*i*)log_2_(*p*(*i*)) (11)
(4) Calculate the frequency-domain cross-correlation coefficient *ρ_i_*:
  (12) $$\rho_{i}=\frac{\int_{0}^{f_{a}}(G_{u_{i}}-{\bar{G}}_{u})(G_{x_{i}}-{\bar{G}}_{x})\mathrm{df}}{\sqrt{\int_{0}^{f_{a}}{\left(G_{u_{i}}-{\bar{G}}_{u}\right)}^{2}\mathrm{df}}\cdot \sqrt{\int_{0}^{f_{a}}{\left(G_{x_{i}}-{\bar{G}}_{x}\right)}^{2}\mathrm{df}}}$$
  where *G_u_* and *G_x_* are the power spectra of the signal *u_i_* and *x*(*t*); ${\bar{G}}_{u}$ and ${\bar{G}}_{x}$ are the corresponding mean values of the power spectra; and *f_a_* is the analysis frequency.
(5) Calculate the time–frequency-domain weighted value of *u_i_*(*t*):
  *θ_i_* = *β*⋅Δ*q_i_* + *γ* ⋅ *ρ_i_* (13)
(6) Calculate the *CEF* based on the time–frequency-domain weighted value:
  (14) $$\delta_{i}=\frac{\theta_{i}-\min(\theta )}{\max(\theta )-\min(\theta )}$$
(7) Calculate the *CEF* of each component and form a descending sequence: {*δ*_1_, *δ*_2_, …, *δ_K_*}.
(8) Calculate the difference between the comprehensive evaluation factors of two adjacent components:
  *d_i_* = *δ_i_* − *δ_i_*_+1_ (15)
(9) Find the index m of the maximum difference. The first m components of the reordered sequence {*δ*} according to the comprehensive evaluation factor are the sensitive modal components containing the main feature information of the signal.

The two components of the CEF characterize fault sensitivity from complementary physical perspectives. The energy entropy increment quantifies the degree to which the occurrence of a fault redistributes the vibration energy among frequency bands: when a localized gear fault (crack, wear or tooth breakage) excites structural resonance, the energy of the corresponding resonance band rises sharply and the energy distribution becomes highly uneven, so the fault-sensitive IMF exhibits a large energy entropy increment, whereas noise-dominated IMFs have dispersed, low-proportion energy and small increments. The frequency-domain cross-correlation coefficient measures how well the power spectrum of an IMF preserves the spectral structure of the original signal, including the meshing frequency, its harmonics and the modulation sidebands; because the power spectrum of random noise is flat and weakly correlated, this coefficient is inherently robust to noise. Therefore, the energy entropy increment captures the fault-induced energy anomaly from an energy perspective, while the frequency-domain cross-correlation captures spectral structure similarity from a frequency perspective, and their fusion in the CEF avoids the one-sidedness of any single criterion.

It is less affected by interference factors during the discrimination process. It can more effectively identify false IMF components that cannot characterize fault information, weaken the influence of modal components unrelated to fault information, and avoid human factor interference caused by threshold setting, thus improving the accuracy of fault feature extraction.

### 2.4. RCMFE Theory

RCMFE is an advanced nonlinear dynamic analysis method developed based on conventional multiscale fuzzy entropy (MFE). The core improvement of RCMFE lies in its adoption of the refined composite multiscale coarse-graining procedure, which not only retains more effective dynamic information embedded in the original time series but also fundamentally addresses the inherent information loss problem caused by the single coarse-graining process in traditional multiscale entropy methods. This refined processing strategy significantly reduces statistical fluctuations and computational inaccuracies, leading to superior measurement accuracy and stability. Consequently, RCMFE exhibits exceptional feature extraction capability for nonlinear and non-stationary vibration signals, which are commonly encountered in mechanical fault diagnosis applications. In this study, RCMFE is directly adopted from [25] without modification.

### 2.5. BP Neural Network Based on SSA Optimization

#### 2.5.1. SSA

Proposed by Xue and Shen in 2020 [32], the SSA is a novel swarm intelligence optimization technique inspired by the collective foraging and anti-predation behaviors of sparrow populations. Compared with traditional optimization algorithms, the SSA exhibits superior global search capability, faster convergence speed, and simpler implementation, making it particularly suitable for solving complex engineering optimization problems such as parameter tuning of neural networks.

In the SSA, the entire sparrow population is divided into three distinct roles with complementary functions: producers (responsible for global exploration and food searching), scroungers (responsible for local exploitation and following producers), and scouts (responsible for danger detection and population alerting). The position of each sparrow in the search space corresponds to a candidate solution to the optimization problem.

Producers are individuals with the highest fitness values in the current population. They have priority in accessing food resources and are responsible for exploring new search areas to guide the entire population. The position update rule for producers is defined as:

(16) $$X_{i,j}^{t+1}=\left\{\begin{array}{l} X_{i,j}^{t}\cdot \exp(\frac{-i}{\Phi \cdot {\mathrm{iter}}_{\max}}),\;\;\;R_{2}<\mathrm{ST}\\ X_{i,j}^{t}+Q\cdot L,\;\;\;\;\;\;\;\;\;\;\;\;\;\;\;\;\;\;\;\;\;R_{2}\geq \mathrm{ST} \end{array}\right.$$

where *t* is the current iteration number, $X_{i,j}^{t}$ is the value of the *j*-th dimension of the *i*-th sparrow at iteration *t*, and *iter*_max_ is the maximum number of iterations. *Φ* ∈ (0,1] is a random number, *R*_2_ ∈ [0,1] is the alarm value, and *ST* ∈ [0.5,1.0] is the safety threshold. *Q* is a random number following a standard normal distribution, and *L* is a 1 × *d* matrix with all elements equal to 1.

When *R*_2_ < *ST*, it indicates that no predators are detected in the current search area, and producers perform extensive global exploration. When *R*_2_ ≥ *ST*, it means that some sparrows have spotted predators, and the entire population needs to quickly move to other safe areas.

Scroungers follow producers to obtain food resources. They continuously monitor the activities of producers and will immediately leave their current positions to compete for food once a high-quality food source is discovered. The position update rule for scroungers is given by:

(17) $$X_{i,j}^{t+1}=\left\{\begin{array}{l} Q\cdot \exp(\frac{X_{w}^{t}-X_{i,j}^{t}}{i^{2}}),\;\;\;\;\;\;\;\;\;\;\;\;\;\;\;\;\;\;\;\;\;\;i<\frac{n}{2}\\ X_{P}^{t+1}+\left|X_{i,j}^{t}-X_{P}^{t+1}\right|\cdot A^{+}\cdot L,\;\;\;\;\;\;\;\;i\geq \frac{n}{2} \end{array}\right.$$

where *X_P_* is the optimal position currently occupied by producers and *X_w_* is the global worst position in the current population. *A* is a 1 × *d* matrix where each element is randomly assigned a value of 1 or −1, and $A^{+}=A^{T}{\left(AA^{T}\right)}^{-1}$.

When *i* > *n*/2, it indicates that the *i*-th scrounger has a poor fitness value and is in a starving state, requiring it to fly to other areas to search for food. Otherwise, the scrounger moves towards the optimal position found by producers to compete for food resources.

Scouts, accounting for 10% to 20% of the total population, are responsible for detecting predators and alerting the flock. Their initial positions are randomly generated within the search space. The position update rule for scouts is expressed as:

(18) $$X_{i,j}^{t+1}=\left\{\begin{array}{l} X_{b}^{t}+\omega \left|X_{i,j}^{t}-X_{b}^{t}\right|,\;\;\;\;\;\;\;\;\;\;\;\;\;\;\;\;\;\;\;\;\;\;f_{i}>f_{g}\;\;\;\;\;\;\;\;\\ X_{i,j}^{t}+k\left(\frac{\left|X_{i,j}^{t}-X_{w}^{t}\right|}{(f_{i}-f_{\omega })+\theta }\right),\;\;\;\;\;\;\;\;\;\;\;f_{i}=f_{g} \end{array}\right.$$

where *X_b_* is the current global optimal position. *ω* is a step size control parameter that follows a standard normal distribution with mean 0 and variance 1. *k* ∈ [−1,1] is a random number that controls both the direction and step size of the sparrow’s movement. *f_i_* is the fitness value of the current sparrow; *f_g_* and *f_w_* are the current global best and worst fitness values, respectively. *θ* is a small constant introduced to avoid division-by-zero errors.

When *f_i_* > *f_g_*, it indicates that the sparrow is located at the edge of the population and is vulnerable to predators, so it moves towards the safe center of the population. When *f_i_* = *f_g_*, it means that the sparrow is in the middle of the population and has detected danger, so it adjusts its position to be closer to other individuals to reduce the risk of predation.

The SSA iteratively updates the positions of producers, scroungers, and scouts according to the above rules until the maximum number of iterations is reached or a predefined convergence criterion is satisfied. The sparrow with the highest fitness value at the end of the iteration is considered the optimal solution to the problem.

#### 2.5.2. BP Neural Networks

The BP neural network is a classic multi-layer feed-forward network, and its learning process consists of forward signal propagation and error back-propagation, through which connection weights and biases are iteratively adjusted to minimize the prediction error. In this study, a two-hidden-layer BP network is constructed for gear fault pattern recognition, where the number of neurons in the two hidden layers is automatically optimized by the SSA described in Section 2.5.3.

#### 2.5.3. SSA-Optimized BP Neural Network

To avoid over-fitting and ensure an unbiased evaluation, the SSA no longer uses the test set error: the classification error on the training and validation subsets (derived from the development samples by five-fold cross-validation) is taken as the fitness function to optimize the number of hidden layer neurons, whereas the held-out test samples are never accessed during this process. The detailed implementation steps are as follows:

Step 1: Initialize the parameter combination [*h*_1_,*h*_2_], define its value range, then initialize the core parameters of the SSA.

Step 2: Calculate the fitness value and corresponding spatial position of each sparrow in the initial population.

Step 3: Update the positions of sparrows based on their fitness: producers update their positions according to Equation (16); scroungers update their positions according to Equation (17); and scouts update their positions according to Equation (18).

Step 4: Recalculate the fitness values of all sparrows in the current population, select excellent individuals as candidates for the next generation population, and update the parameter combination

Step 5: Determine whether the maximum number of iterations is satisfied. If yes, output the optimal number of hidden layer neurons; otherwise, return to Step 2 for iterative optimization until the stopping condition is met.

**Figure 3:**
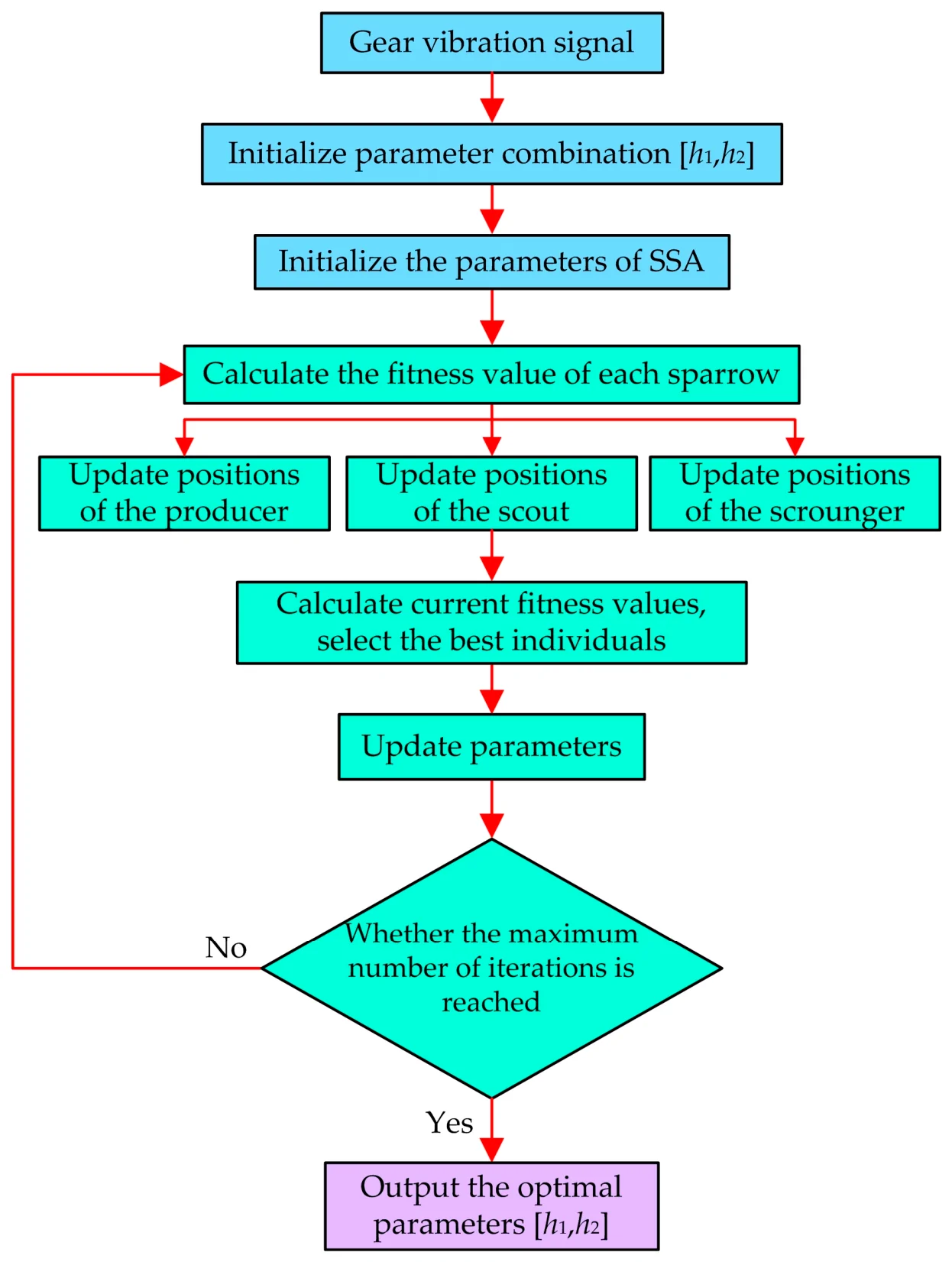
Flowchart of the SSA-optimized BP neural network.

A flowchart of the SSA-optimized BP neural network is shown in Figure 3.

## 3. The Proposed Fault Diagnosis Method Based on COA-VMD, RCMFE and SSA-BP Neural Network

This paper combines the effectiveness of COA-VMD in signal decomposition, the superiority of RCMFE in signal feature extraction and the accuracy of the SSA-BP neural network in state classification to propose a gear fault diagnosis method.

It should be emphasized that the COA, the SSA and RCMFE are established algorithms that are adopted in this work; the new mathematical and methodological contributions are (i) the composite index CI fusing permutation entropy and mutual information entropy for label-free VMD parameter optimization and (ii) the comprehensive evaluation factor CEF combining the energy entropy increment with the frequency-domain cross-correlation coefficient for automatic sensitive IMF selection, together with (iii) their systematic integration into a complete gear fault diagnosis framework.

**Figure 4:**
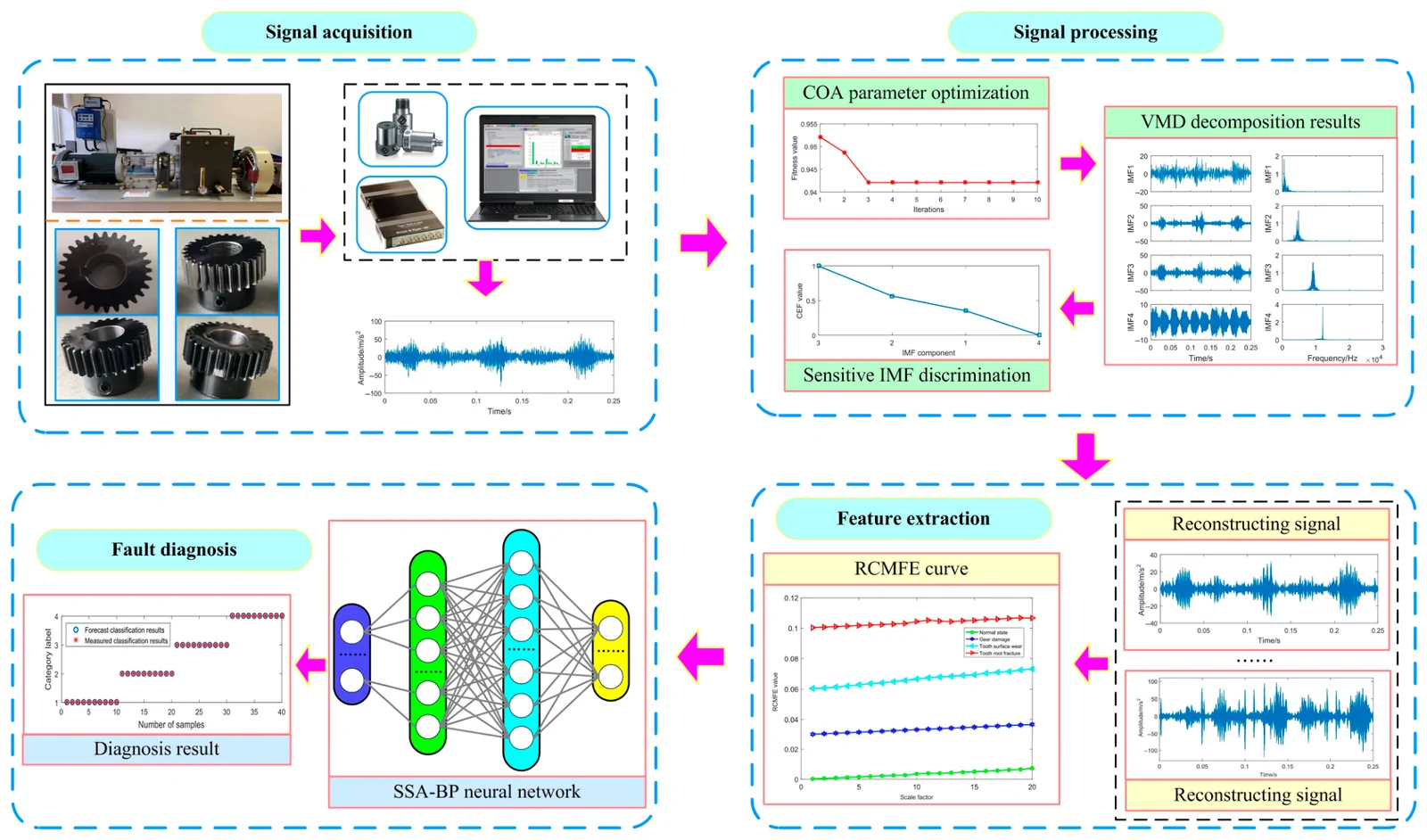
Flowchart of the gear fault diagnosis method based on COA-VMD, RCMFE and SSA-BP.

The diagnostic flow is shown in Figure 4, and the pseudo-code (Algorithm 1) describes the gear fault diagnosis process; its main steps are as follows:

Step 1: Collect gear vibration signals under different working conditions on rotating mechanical equipment.

Step 2: For gear vibration signals under different states, take the composite index combining PE and MIE as the fitness function, use the COA to optimize the key parameters of VMD, and decompose the relevant signals using the optimized VMD to obtain a series of IMF components.

Step 3: Calculate the time–frequency-domain comprehensive evaluation factor of each IMF component, and select the components sensitive to signal features for signal reconstruction based on this factor.

Step 4: Use RCMFE to extract the features of the reconstructed signal and form a state feature set.

Step 5: Take the classification error on the training and validation subsets as the fitness function, use the SSA to optimize the number of hidden layer neurons, and keep the independent test set completely unseen until the network structure is fixed; then, input the state feature set into the determined SSA-BP neural network to diagnose the gear working conditions.
  **Algorithm 1** COA-VMD, RCMFE and SSA-BP gear fault diagnosis method  Input: gear vibration signals under C fault categories  Output: predicted fault category  01: **for** each fault category **do**  02: initialize the COA population and the VMD search ranges *K* ∈ [2,7], *α* ∈ [100,5000]  03: **while** stopping criterion is not met **do**  04: **for** each coati individual, run VMD and compute the composite index CI = f(PE, MIE)  05: **end for**  06: update positions in the hunting (exploration) and escape (exploitation) phases  07: **end while**  08: [K*,α*] ← best solution; decompose the signal with the optimized VMD  09: **for** each IMF, compute the energy entropy increment and frequency-domain cross-correlation, then the CEF  10: **end for**  11: select fault-sensitive IMFs by the maximum CEF difference and reconstruct the signal  12: compute the RCMFE over scales *τ* = 1–20 to form a feature vector  13: **end for**  14: construct the feature set; initialize the SSA and the two-hidden-layer BP  15: split the development set into training/validation; optimize [*h*_1_,*h*_2_] by SSA (fitness: train + validation error)  16: train the SSA-BP on the training set and predict the test samples  17: after all parameters are fixed, predict the untouched test samples

## 4. Experimental Design

To comprehensively verify the effectiveness of the proposed method, experimental analyses are conducted on both gear single fault and compound fault signals. To highlight the superiority of VMD in nonlinear signal processing and the effectiveness of COA-based key parameter selection, comparative methods based on EMD-RCMFE, EEMD-RCMFE, complete ensemble empirical mode decomposition with adaptive noise (CEEMDAN)-RCMFE and default parameter VMD-RCMFE are established. To demonstrate the excellent performance of the SSA-BP classifier, three classic machine learning methods, including a BP neural network, KNN and DT, are selected for comparison of classification accuracy. For each fault type, 20 samples are completely held out as the independent test set, and the remaining 60 samples form the development set, from which training and validation subsets are derived using five-fold cross-validation (equivalently 48 training and 12 validation samples per fold) for network structure and hyperparameter selection. SSA optimization uses only the training/validation error, whereas the test samples remain unseen until all preprocessing choices and model parameters have been fixed. Each experiment is repeated 10 times with independent data splits, and the mean and standard deviation of the test results are reported.

For clarity, the data organization and the input/output tensor dimensions are specified as follows. After COA-VMD reconstruction, the RCMFE values over scales τ = 6–20 form a 15-dimensional feature vector for each sample. For the single fault experiment, the input feature matrices of the training and test sets are of size 240 × 15 and 80 × 15, respectively, and the corresponding one-hot label tensors are 240 × 4 and 80 × 4 for the four states. For the compound fault experiment, the training and test input matrices are 180 × 15 and 60 × 15, with one-hot label tensors of 180 × 3 and 60 × 3 for the three states. The classifier is a two-hidden-layer BP network, whose optimized structures are 15–33–16–4 for the single fault data and 15–30–48–3 for the compound fault data, where the last dimension denotes the number of fault categories.

### 4.1. Single Fault Experiment

**Figure 5:**
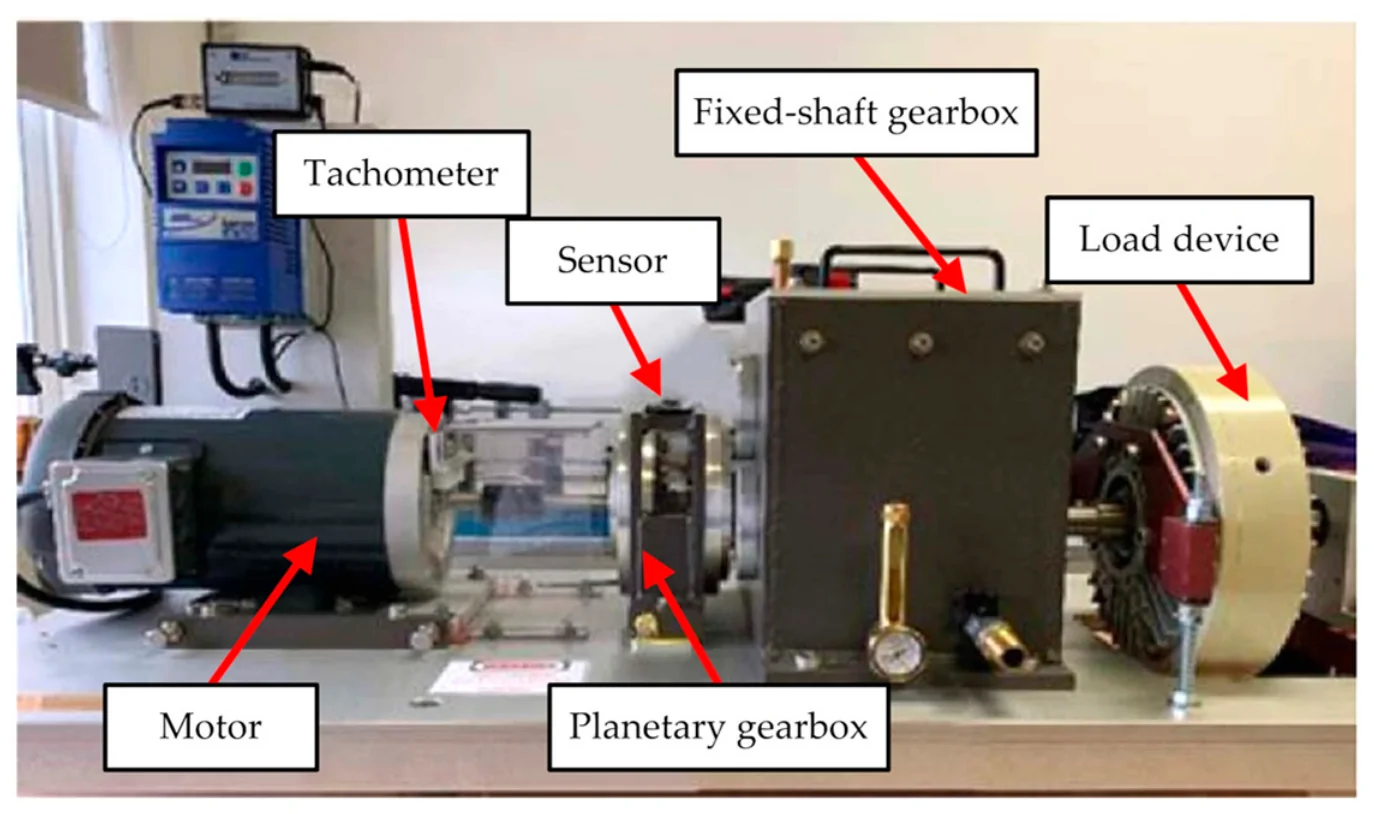
Single fault data acquisition test bench.

The single fault data are taken from the publicly available WT planetary gearbox dataset [33]. The single fault experiment uses the vibration test bench shown in Figure 5, which consists of a motor, a planetary gearbox, a fixed-axis gearbox and a load device. Vibration data is collected by a Sinocera CA-YD-1181 accelerometer (sourced from Sinocera Piezotronics, Inc., Yangzhou, China.), and speed pulses are captured using an encoder with a sampling frequency of 48 kHz. Signals under four states are collected: normal state, crack occurs in the root, worn gear and gear with a broken tooth.

The planetary gear train comprises a 28-tooth sun gear, a fixed 100-tooth ring gear, and four 36-tooth planet gears. The gear meshing frequency is derived as (175/8)*f_r_*, and the characteristic fault frequency of the sun gear is (25/8)*f_r_*, where *f_r_* represents the rotational frequency of the sun gear. The dataset encompasses eight constant rotational speeds of the sun gear (20, 25, 30, 35, 40, 45, 50 and 55 Hz), among which 20 Hz is selected for the present study. All experimental vibration signals are acquired under steady constant speed operating conditions. For each health condition, vibration signals are recorded for no less than 5 min. To account for installation effects, data acquisition is performed twice with full disassembly and reassembly of the gearbox between tests, producing independent experimental runs to evaluate the impact of assembly variations.

**Figure 6:**
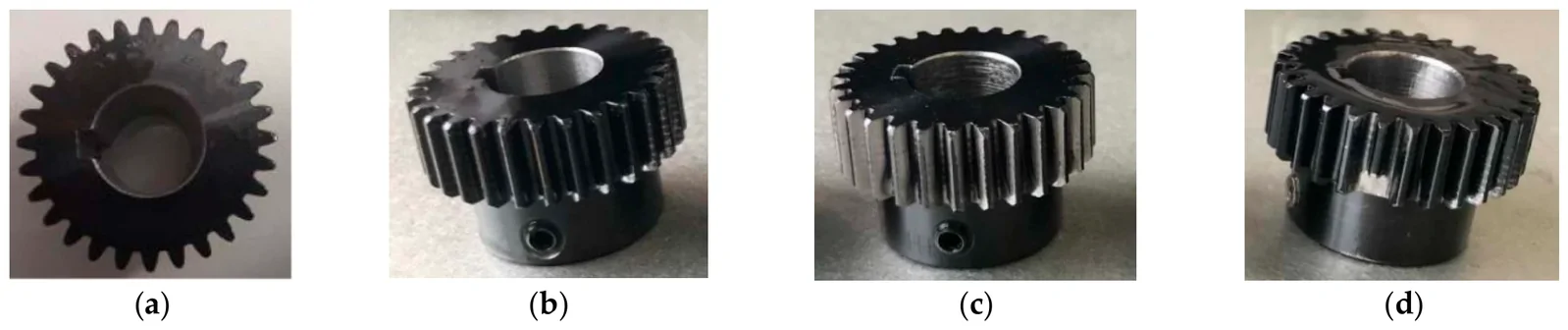
Gears in different states: (**a**) normal state, (**b**) crack occurs in the root, (**c**) worn gear, and (**d**) gear with a broken tooth.

The gears in different states are shown in Figure 6. For each state, 80 samples are selected, each with a length of 12,000 data points. The gear state descriptions are shown in Table 1.

The samples are obtained by cutting the continuous recordings with a fixed-length, non-overlapping sliding window of 12,000 points, and the train/validation/test split is carried out at the run level so that segments from the same physical run never appear in different subsets. All the algorithms are implemented in MATLAB R2016b on a computer equipped with an Intel Core i5-10400 CPU (2.90 GHz), 16 GB RAM and Windows 10, without GPU acceleration.

### 4.2. Compound Fault Experiment

**Figure 7:**
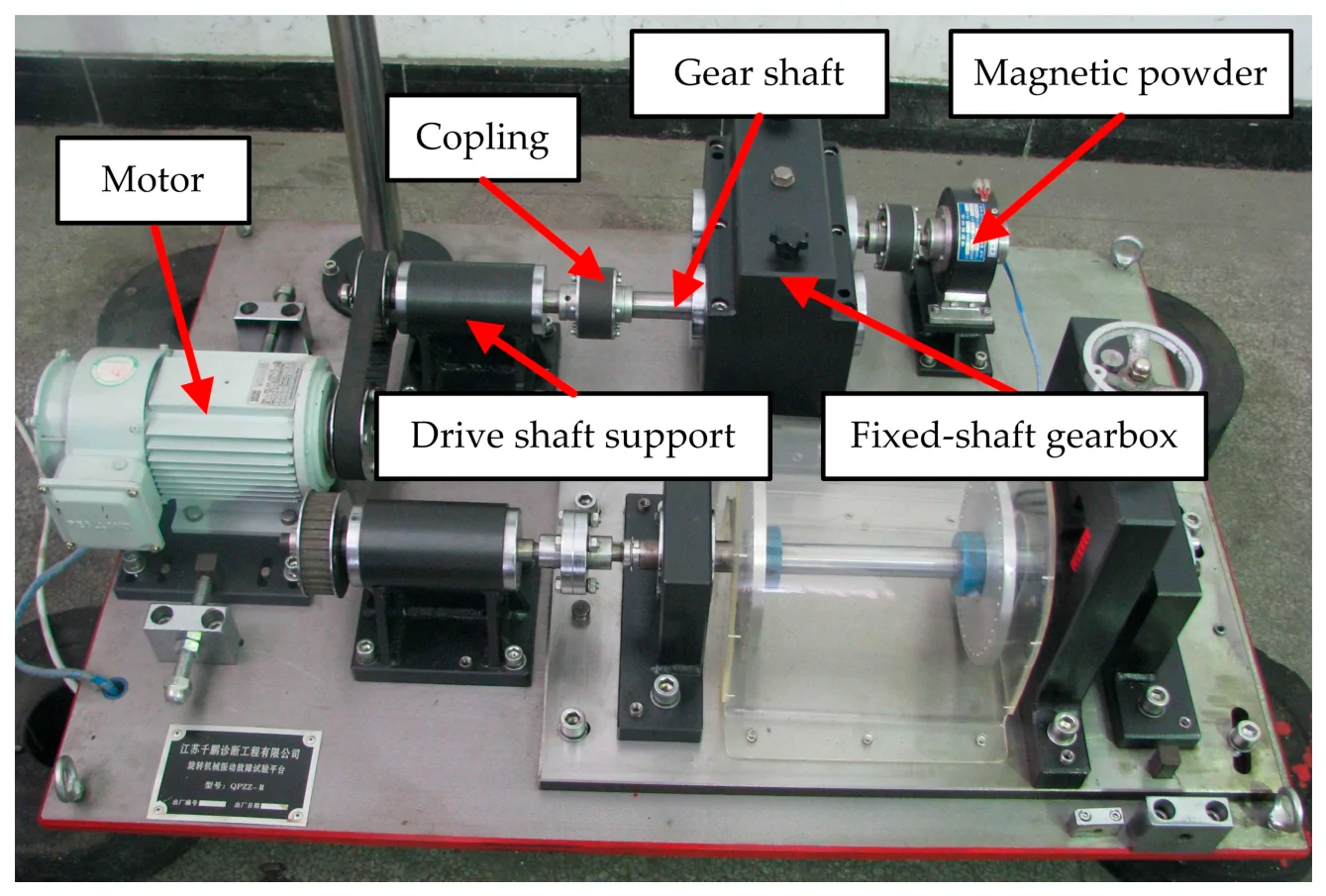
Compound fault data acquisition test bench.

The fault simulation adopts the QPZZ-II test platform shown in Figure 7, which is mainly composed of a drive motor, gear shafts, rotating shafts and couplings. During signal acquisition, the accelerometer is installed on the bearing seat of the gearbox, and an ADA16-8/2 (LPCI) data acquisition card is used with a sampling frequency of 5120 Hz and a rotating speed of 1470 r/min.

**Figure 8:**
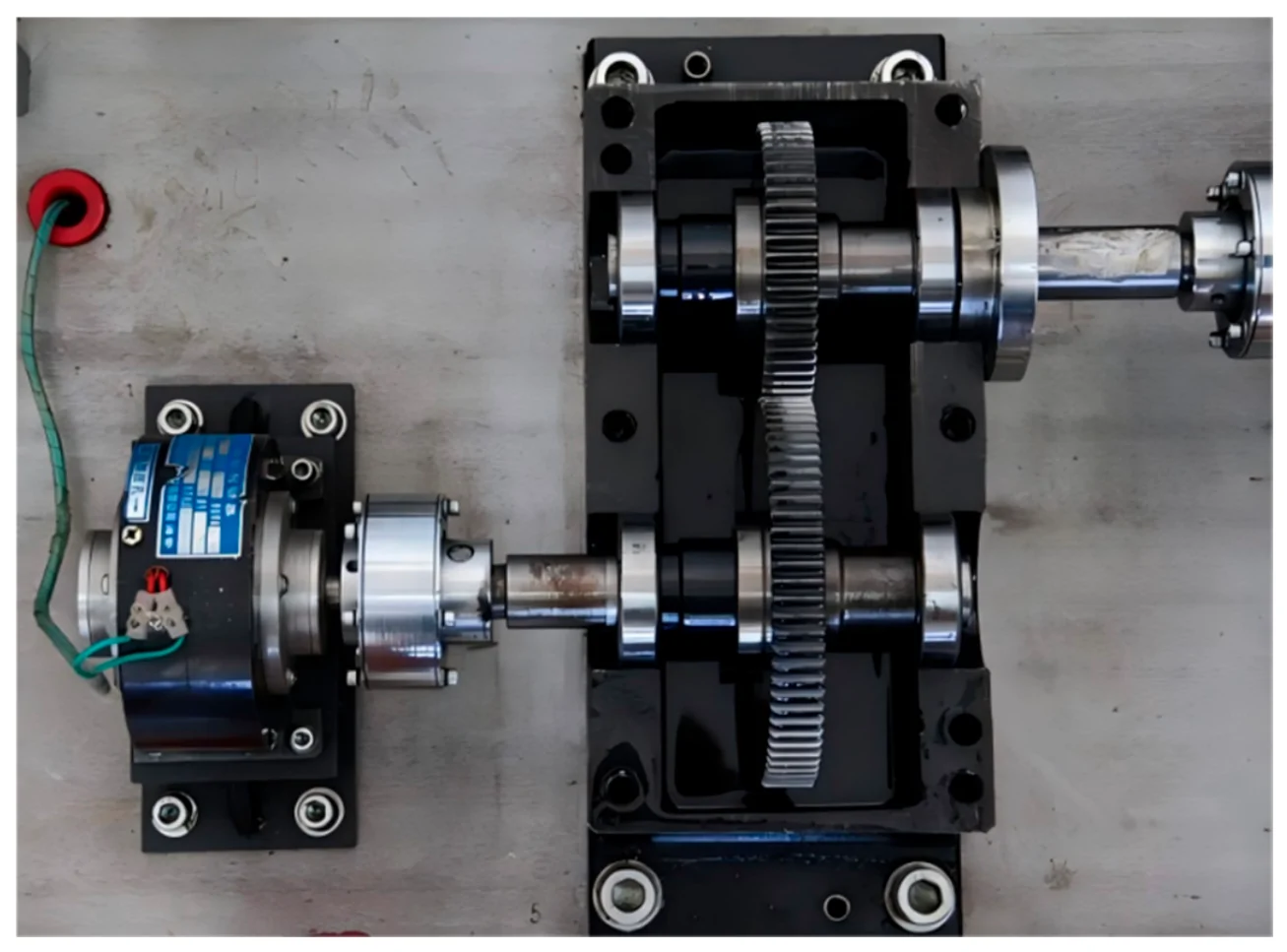
Internal structure of gearbox.

The gearbox of the test platform mainly consists of a driving gear with 55 teeth and a driven gear with 75 teeth. Both gears are made of S45C steel and are spur cylindrical gears. The internal structure of the gearbox is shown in Figure 8. The driving gear is used to simulate wear faults, mainly single-tooth surface wear with artificial grinding, and the wear degree is 15% of the tooth thickness. The driven gear is used to simulate tooth breakage and pitting faults: the tooth breakage fault is that one tooth of the driven gear is broken, with the fracture line located near the pitch circle; the pitting fault is that the tooth surface of the driven gear is spot-welded to form pits with an area ratio of 2%, simulating moderate pitting.

Signals under normal, tooth breakage-wear and pitting-wear compound fault states are collected. For each state, 80 samples are selected, each with a length of 1280 data points. The gear state descriptions are shown in Table 2.

Likewise, the compound fault samples are cut from continuous recordings using non-overlapping 1280-point windows; each state is recorded in several independent runs, and the run-level split prevents segments of the same physical run from entering different subsets. Sampling is performed at 5120 Hz with a 2 kHz anti-aliasing cutoff at a constant speed of 1470 r/min.

## 5. Results and Analysis

### 5.1. Single Fault Diagnosis

**Figure 9:**
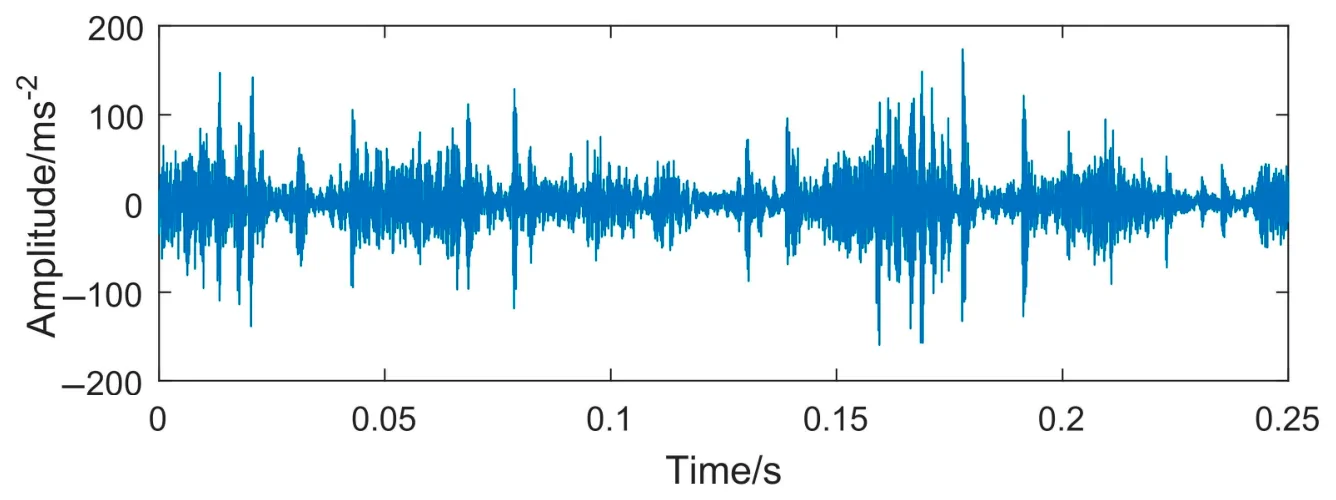
Time-domain waveform of the crack in the root fault signal.

The data with labels 1, 2, 3 and 4 are used for the experiment. Taking the crack occurring in the root fault data with label 2 as an example, a signal is randomly selected for analysis, and its time-domain waveform is shown in Figure 9.

**Figure 10:**
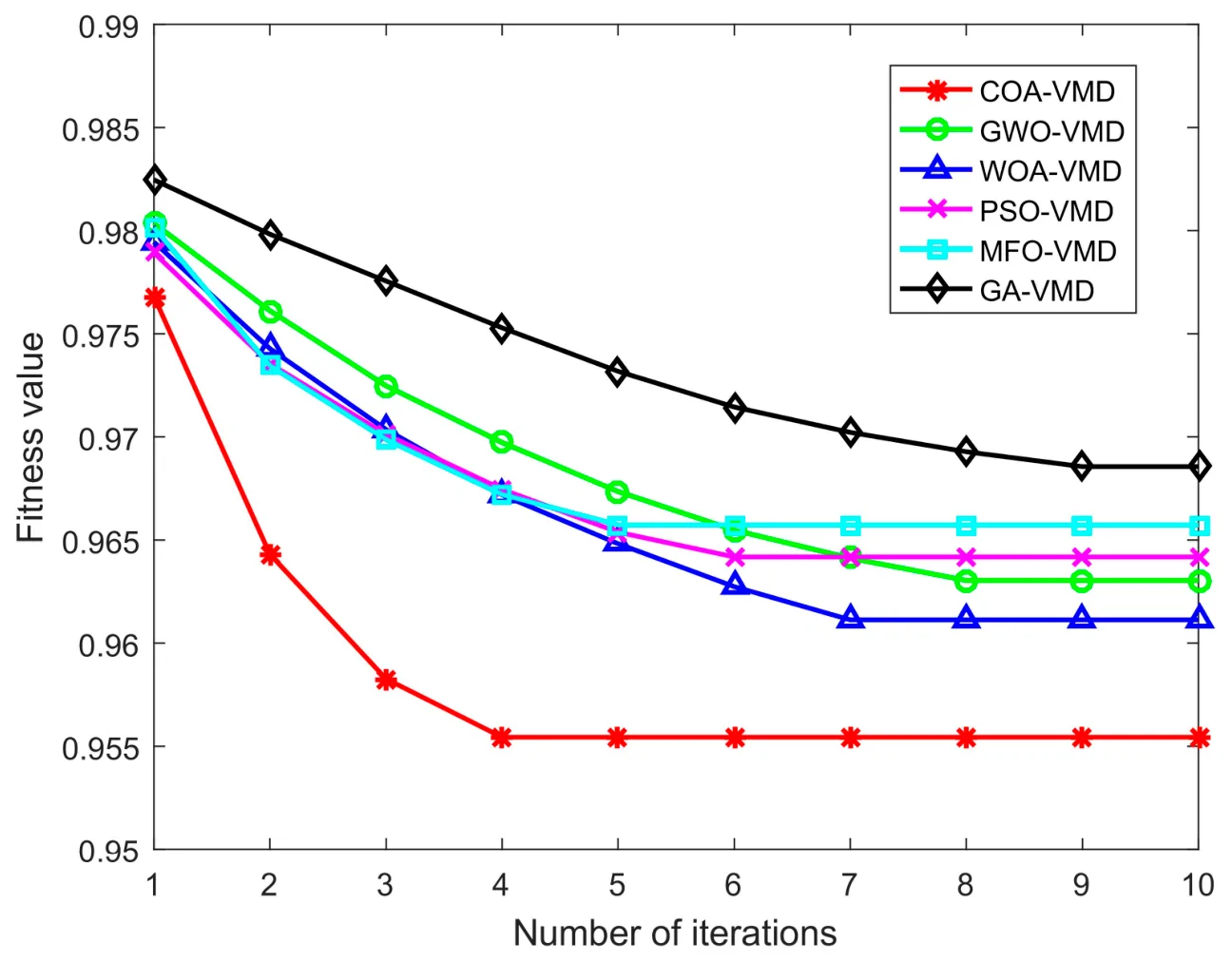
Iterative curve of fitness.

It can be seen from Figure 9 that the collected signal contains a large amount of noise due to the lack of a noise filtering device. If features are extracted directly from this signal, it will be difficult to obtain effective information. Therefore, VMD is used to decompose the signal to filter out the influence of noise interference components. To achieve accurate signal decomposition, the COA is used to optimize the two key parameters of VMD. Referring to references [10,34], with the CI as the fitness function, the optimization is performed within the ranges of *K* ∈ [2,7] and *α* ∈ [100,5000]. To demonstrate the excellent search performance of the COA, GWO, the WOA, particle swarm optimization (PSO), moth–flame optimization (MFO) and the genetic algorithm (GA) are selected for comparison. In the experiment, the population size of each optimization algorithm is set to 50, and the maximum number of iterations is 10. The above methods are run 10 times to optimize the VMD parameters of the crack occurring in the root fault signal in Figure 9. The optimal fitness iteration curves and optimization results (including the mean ± standard deviation of 10 runs) are shown in Figure 10 and Table 3, respectively.

Because all optimizers are stochastic, the conclusions are based on 10 independent realizations rather than a single run. Over the 10 runs, COA-VMD attains best/worst fitness of 0.95544/0.95600 with the smallest standard deviation, while the corresponding best/worst values are 0.96113/0.96287 for the WOA, 0.96304/0.96476 for GWO, 0.96418/0.96622 for PSO, 0.96573/0.96787 for MFO and 0.96856/0.97164 for the GA. A two-sided Wilcoxon rank-sum test between the 10 fitness values of the COA and those of each competitor returns *p*-values below 0.01 in every case, confirming that the superiority of the COA is statistically significant and is not due to a favorable single realization.

It can be seen from Figure 10 that the COA achieves convergence at the fourth iteration, which is faster than the other optimization algorithms. Meanwhile, Table 3 shows that COA-VMD obtains the best fitness value and mean ± standard deviation.

The advantage of the COA over other optimization algorithms can be understood from its search mechanism. In GWO, the population is guided by three dominant wolves through an encircling operation, which is efficient but prone to premature convergence when the leaders are trapped in local optima. The WOA updates individuals using a spiral bubble-net strategy, in which the balance between exploration and exploitation is controlled mainly by a single coefficient vector, providing limited population diversity. PSO relies on personal and global best positions with velocity inertia and may oscillate in high-dimensional multimodal spaces; MFO drives moths toward flames along logarithmic spirals, but the number of guiding flames is reduced monotonically until only the single best flame remains, which sharply weakens population diversity in the later iterations and makes the moths prone to swarming around a locally optimal flame and converging prematurely; the GA depends strongly on crossover and mutation settings and converges slowly near the optimum. In contrast, the COA explicitly separates global exploration and local exploitation into two phases: in the hunting phase, two cooperating subgroups and the random repositioning of the iguana within the search space maintain high population diversity and avoid local optima; in the escape phase, dynamically shrinking local search bounds combined with greedy selection perform fine-grained exploitation around promising solutions. This explicit two-phase balance gives the COA stronger global search capability and faster, more stable convergence; on a wide range of unimodal, multimodal and composite benchmark functions, as well as constrained engineering design problems, the COA has been reported to outperform GWO, the WOA, MFO, PSO and the GA, which motivates its selection for VMD parameter optimization in this study.

**Figure 11:**
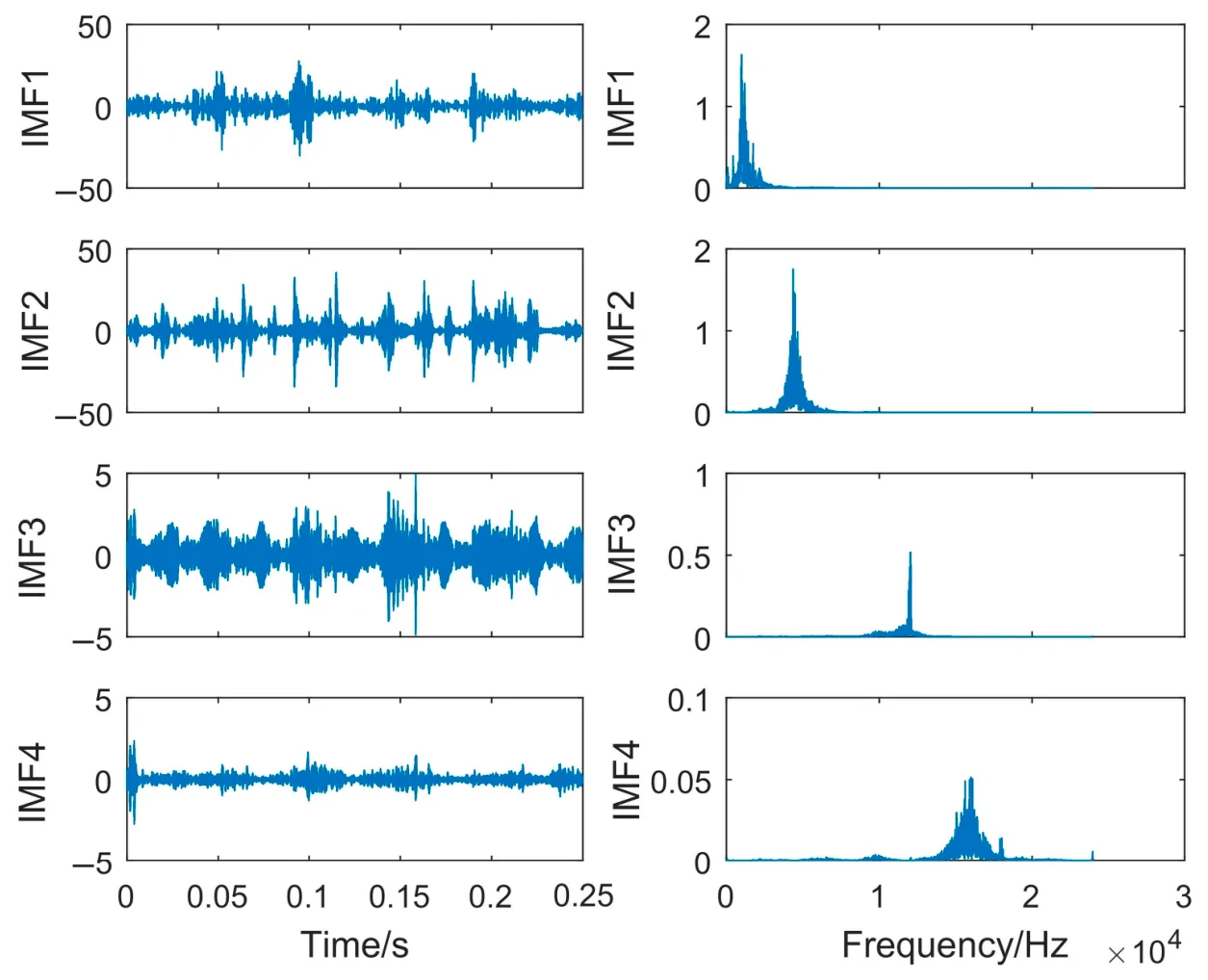
Decomposition of the crack occurs in the root fault signal.

The time-domain waveforms and spectra of the IMF components obtained by decomposing the signal in Figure 9 using COA-VMD are shown in Figure 11.

It can be seen from Figure 11 that VMD is reasonable, and each IMF component is mainly concentrated near its center frequency without obvious frequency divergence. This indicates that the proposed parameter selection method can effectively suppress the mode aliasing problem generated during the decomposition process, reduce information leakage between modal components, and provide a guarantee for subsequent signal feature extraction, thus verifying the effectiveness of the COA in optimizing VMD key parameters.

COA-VMD parameter optimization is performed on the data with labels 1, 2, 3 and 4 in sequence, and the obtained optimal parameter combinations are shown in Table 4.

An important point for practical deployment is that this optimization does not use any fault label. The composite index CI is computed only from the signal itself (permutation entropy of the IMFs and mutual information with the original signal), so it is an unsupervised criterion. In real diagnosis, the VMD parameters are determined once from the unlabeled development signals and then fixed; every new unlabeled test signal is decomposed with these fixed parameters without knowing its fault class. The state-specific values in Table 4 are reported only as an illustrative analysis, revealing how the optimal K and α vary with signal complexity and impact content, and are not used to select parameters at test time. Alternatively, because the CI needs no label, it can also be minimized independently for each new signal without creating any class information circularity.

The variation in the optimal *K* and *α* across fault states can be explained by the physical meaning of the VMD parameters and the spectral characteristics of the fault signals. *K* determines the number of distinct frequency bands contained in the signal, while *α* controls the bandwidth penalty, with a larger *α* forcing narrower, more independent bands. In the normal state, the signal is dominated by the meshing frequency and its regular harmonics, giving a simple and stable spectral structure. A root crack generates periodic impacts that excite a concentrated high-frequency structural resonance; because this resonance band is narrow and well defined, a larger α (α = 3736) with a smaller *K* (*K* = 4) is preferred to keep it independent of adjacent components. A broken tooth produces stronger impacts with richer harmonics and energy spread over more bands, requiring the largest number of modes (*K* = 6). Wear mainly introduces additional modulation sidebands around the meshing harmonics, corresponding to *K* = 4 and an intermediate α (*α* = 2221). This fault-dependent variation is not a deficiency but rather evidence supporting adaptive parameter optimization: any fixed K and α would under- or over-decompose part of the fault signals, whereas COA-VMD automatically matches the decomposition structure to each individual fault signal.

**Figure 12:**
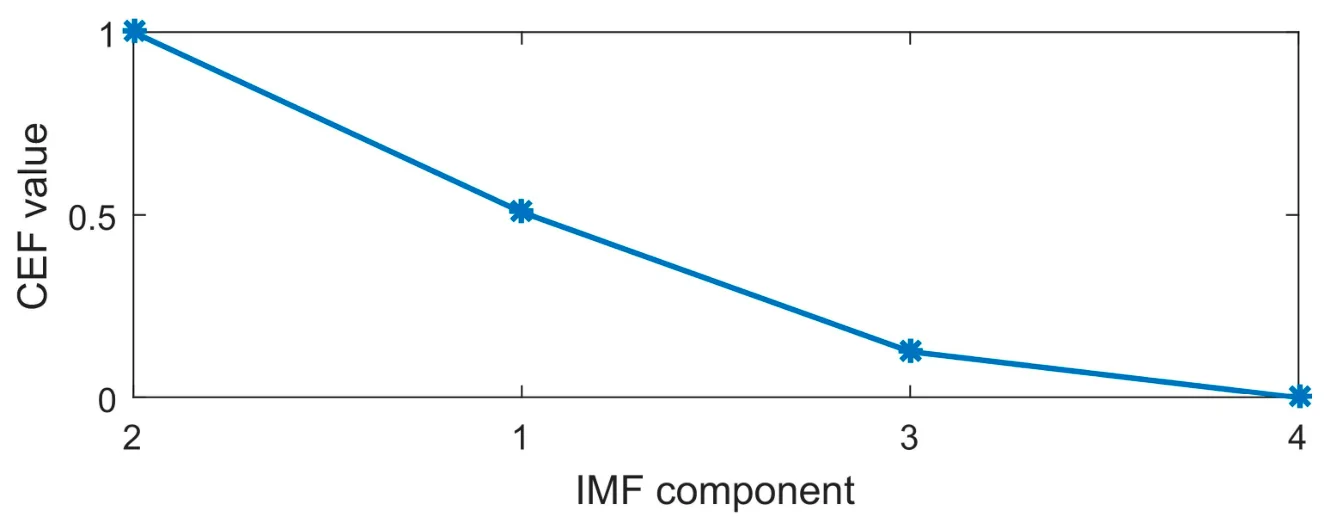
Comprehensive evaluation factors of IMF components.

When the frequency components of the signal change rapidly, time-domain analysis may not accurately capture the signal characteristics, while frequency-domain analysis can more comprehensively reveal the frequency components contained in the signal. False modal components usually have unique frequency-domain characteristics, such as sharp peaks or abnormal frequency components. Therefore, frequency-domain information is more critical than time-domain information in false IMF component discrimination. Based on the experimental results in Section 5.3, *β* = 0.4 and *γ* = 0.6 are used in Equation (9). The time–frequency-domain comprehensive evaluation factors of each IMF component shown in Figure 11 are calculated and arranged into a descending sequence {*δ*}, as shown in Figure 12.

**Figure 13:**
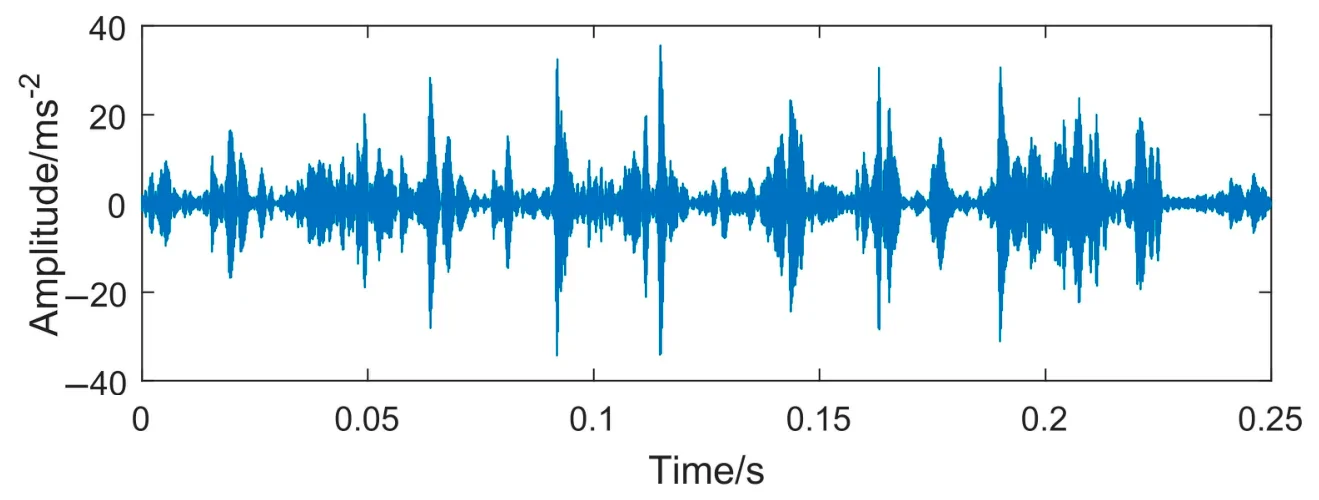
Time-domain waveform of the reconstruction signal.

It can be seen from Figure 12 that the maximum difference occurs between IMF2 and IMF1. IMF2 is the sensitive IMF component containing the fault features, while IMF1, IMF3 and IMF4 are interference components containing noise and background signals. Therefore, IMF2 is selected as the sensitive IMF component for reconstruction, and the time-domain waveform of the reconstructed signal is shown in Figure 13.

Comparing Figure 9 and Figure 13, it can be seen that the proposed method effectively filters out most of the interference components such as noise, enhances the characteristics of the fault signal, and highlights the impact component of the signal, which provides support and guarantees for the subsequent fault feature extraction.

To effectively extract signal features, the RCMFE values of the reconstructed signals are calculated. When calculating RCMFE, important parameters such as embedding dimension *m*, fuzzy function gradient *n*, similarity tolerance *r* and scale factor *τ* need to be preset. The selection of these parameters has a crucial impact on the calculation results of RCMFE and directly relates to the accuracy and reliability of the analysis results.

For the selection of embedding dimension *m*, there are generally two considerations. First, from the perspective of data characteristics, if the internal structure of the data is complex and there are various fluctuation patterns, a larger embedding dimension is needed to fully capture its dynamic characteristics. For example, when analyzing complex electroencephalogram signals containing various rhythms, a larger embedding dimension is required to reconstruct the phase space and better reveal the potential laws in the signal. Second, from the perspective of practical application, if the research purpose is state classification, a smaller embedding dimension of two or three can meet the requirements [35,36]. To distinguish several working states of mechanical equipment, a low dimension can not only meet the requirements but also reduce the computational complexity and time. A larger embedding dimension will significantly increase the computational complexity and time. Therefore, within the limits of available computing resources, the embedding dimension *m* is selected as two.

If the fuzzy index *n* is too large, the membership function curve will become steep. On the one hand, this will cause the system to be overly sensitive to small changes in input; on the other hand, an excessively large *n* may make the boundary division of fuzzy sets too strict, resulting in some data that should have a certain fuzzy membership being unreasonably excluded, thus losing valuable information. Conversely, if *n* is too small, the membership function curve will be too flat, which may make the distinction between fuzzy sets not obvious enough and slow down the system’s response to input changes, leading to degraded system performance. Considering that the gear vibration data collected in this paper was decomposed and reconstructed by the COA-VMD method, and the reconstructed signal contains certain fault feature information, and referring to relevant literature [35], *n* is selected as 2.

The selection of similarity tolerance *r* is usually related to the standard deviation SD of the time series. Generally, *r* is often taken as 0.1 to 0.25 times the standard deviation of the time series. If *r* is too small, the number of similar vectors required for entropy calculation will be too small, which may underestimate the complexity of the system and miss other valuable feature information. On the contrary, if *r* is too large, too many vectors will be judged as similar, thus overestimating the complexity of the system and affecting the judgment of the real state of the system. Therefore, *r* is selected as 0.2 SD in this paper.

There is no absolute good or bad method for the selection of the scale factor *τ* in RCMFE, and it should be selected according to the specific situation. For example, when selecting a small scale factor of 2 to 5, the detailed information of the original data can be better retained, which is very helpful for studying local and rapidly changing dynamic characteristics in some sequences. A large scale factor *τ* > 10 focuses more on capturing the macro trend and long-period changes in the data and can better extract the global correlation in the data. Meanwhile, a larger scale factor can also reduce the influence of noise to a certain extent. As *τ* increases, the noise components in the signal are relatively averaged or weakened, making the evaluation of the essential characteristics of the data more accurate. The above two advantages greatly help the gear fault feature extraction link: first, they reduce the influence of noise on feature extraction; second, they can effectively distinguish different working states of gears by observing the global correlation of different working states under different scale factors. Therefore, *τ*_max_ = 20 is selected in this paper to better observe the global correlation of different working states of gears.

**Figure 14:**
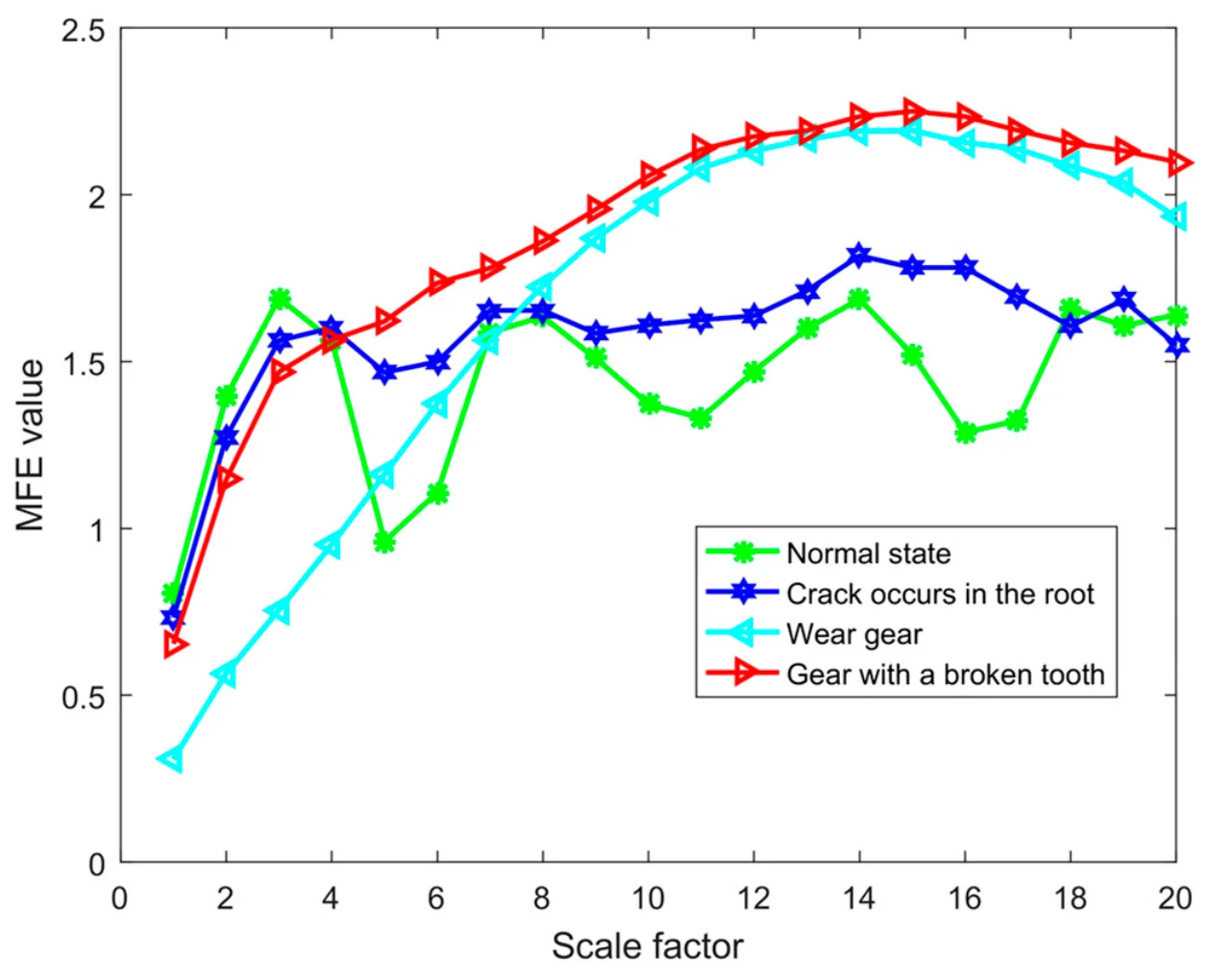
MFE values of single fault reconstructed signals.

**Figure 15:**
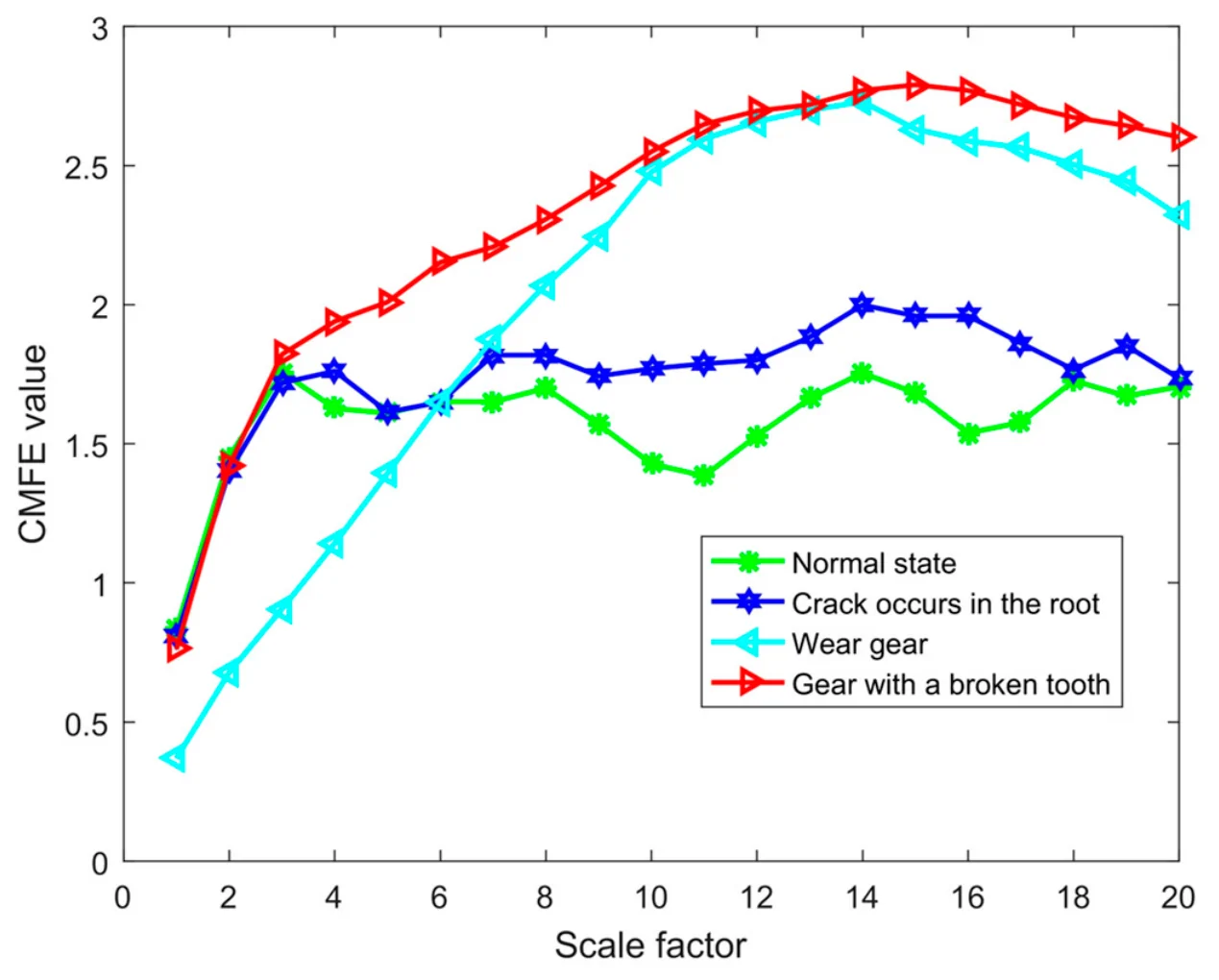
CMFE values of single fault reconstructed signals.

**Figure 16:**
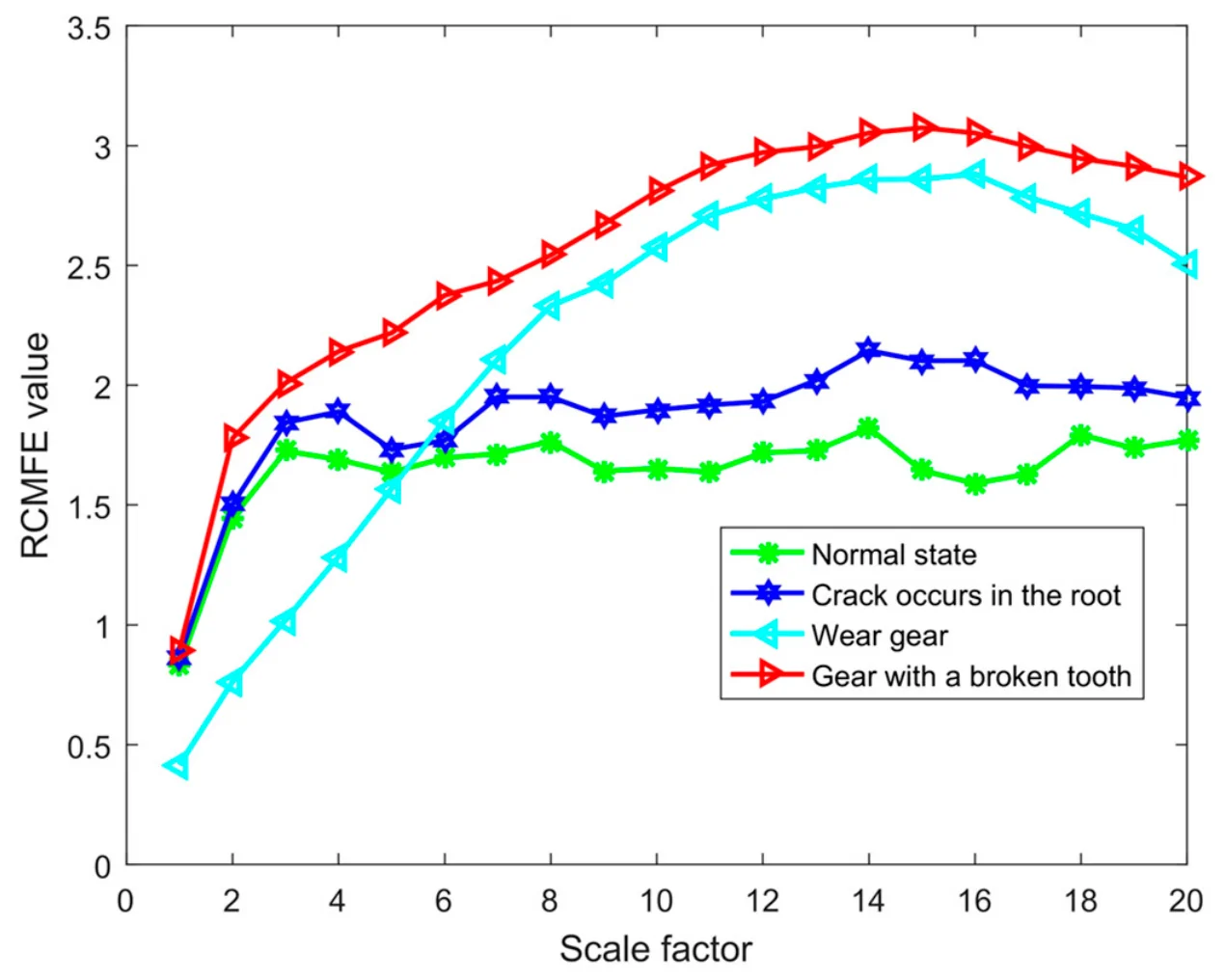
RCMFE values of single fault reconstructed signals.

With embedding dimension *m* = 2, fuzzy index *n* = 2, similarity tolerance *r* = 0.2 SD and scale factor *τ* = 20, the vibration signals with labels 1, 2, 3 and 4 are reconstructed by COA-VMD according to the method in this paper, and the RCMFE values of the reconstructed signals under the four working conditions are calculated. To demonstrate the accuracy of RCMFE in feature extraction, composite multiscale fuzzy entropy (CMFE) and MFE are used for comparative analysis of feature extraction, and the relevant parameters are selected using the same method as RCMFE. A group of reconstructed signals based on COA-VMD is randomly selected, and their MFE, CMFE and RCMFE values are calculated. The results are shown in Figure 14, Figure 15 and Figure 16.

It can be seen from Figure 14 that the MFE curves of single fault reconstructed signals generally show a trend of first rising and then falling. Meanwhile, the entropy value curves have poor stability and many cross-overlapping phenomena, resulting in unsatisfactory fault feature extraction. The main reason is that MFE adopts a single-start coarse-grained averaging decomposition method, which only reconstructs the sequence from the first sampling point in each scale window, losing most of the time-domain information in the window. Moreover, as the scale factor increases, the length of the reconstructed sequence decreases sharply, leading to a significant increase in the statistical variance of entropy estimation and severe fluctuations in the curves. For non-stationary signals such as gears containing a large number of impact modulation components, the feature information of different fault states is seriously confused during the coarse-grained averaging process, which finally manifests as a large number of cross-overlaps between curves of different states.

It can be seen from Figure 15 that after further optimization by the CMFE method, the stability of the entropy value curves is greatly improved, the fluctuation degree is significantly reduced, and the inter-class distance is increased compared with MFE. However, there are still many local cross phenomena in the entropy value curves, and the feature extraction capability needs to be further improved. This is because CMFE uses multi-start composite coarse-grained decomposition to calculate the average entropy value using reconstructed sequences from all starting points, which effectively reduces the variance of entropy estimation, thus significantly improving the smoothness and stability of the curves. Nevertheless, this method still adopts equal-interval coarse-grained division and cannot capture the fine fluctuation characteristics within scales. Moreover, there is still a certain systematic deviation in entropy calculation for strong nonlinear impact signals generated by gear tooth breakage and wear, leading to local cross-overlaps between curves of different states.

It can be seen from Figure 16 that the RCMFE values of the four different gear vibration signals generally show a trend of first rising and then slowly decreasing as the scale factor increases. However, the entropy value curves have better separability and stronger stability, and the cross-overlapping phenomenon is effectively suppressed. The four states are well distinguished, indicating good performance in signal fault extraction and fault discrimination. This is because, on the basis of CMFE, RCMFE introduces a refined composite multiscale decomposition strategy, which performs non-omission sequence reconstruction for all sampling points in each coarse-grained window, completely retaining the time-domain detail information at different scales. Meanwhile, the random error of entropy estimation is further compressed through multi-sequence averaging, greatly improving the stability of features. This method can accurately characterize subtle differences in signal nonlinear complexity under different gear fault states and effectively amplify the signal complexity changes caused by faults, thus achieving effective distinction of the four state curves and possessing the optimal feature extraction performance.

Therefore, the applicability and superiority of the proposed refined composite multiscale fuzzy entropy method in the gear fault diagnosis feature extraction link are verified.

Based on the superiority of the RCMFE method shown in Figure 16 for gear fault feature extraction, it is necessary to select entropy values within an appropriate scale factor range to construct a suitable fault feature vector set. If too many scale factors are selected, the set will contain too much useless information, resulting in information redundancy; if too few are selected, it is easy to lose valuable information, and the set cannot fully reflect the fault characteristics [37]. Therefore, considering the separability of the RCMFE entropy value curves comprehensively, the RCMFE values of the gear reconstructed signals when *τ* = 6 ~ 20 are selected as signal features to form state feature vectors and constitute the state feature set.

**Figure 17:**
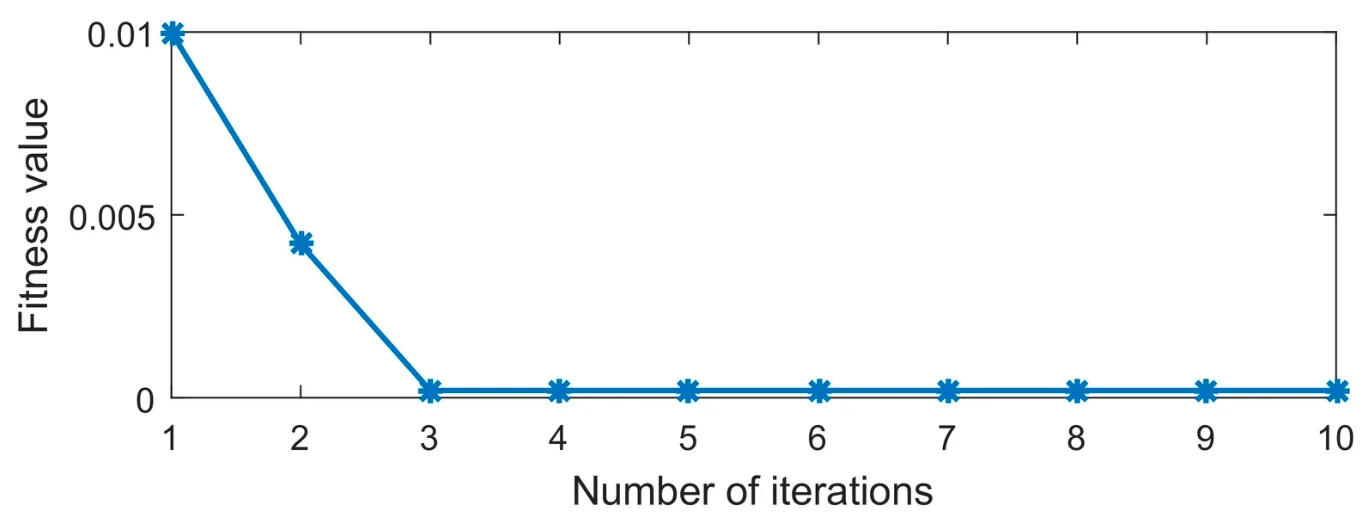
The fitness iteration curve of SSA-optimized BP.

The number of hidden layers of the BP neural network is set to two, the maximum number of iterations is 500, the learning rate is 0.01, and the minimum training target error is 1 × 10^−4^. Referring to references [38,39], the number of hidden layer neurons [*h*_1_,*h*_2_] ∈ [5,50] is optimized using the SSA. In the experiment, the population size of the SSA is 50, and the maximum number of iterations is 10. The fitness iteration curve is shown in Figure 17.

**Figure 18:**
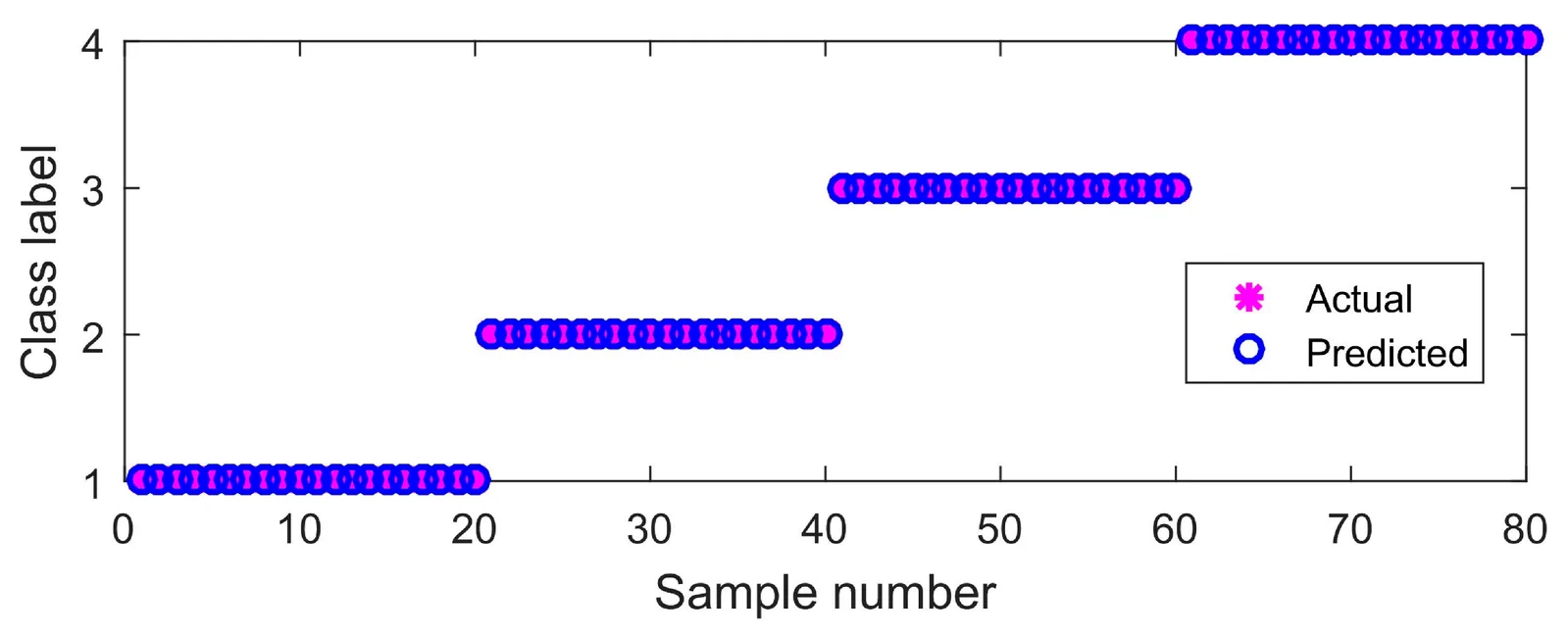
The identification results of single faults.

The optimization result of SSA for the number of BP hidden layer neurons is [*h*_1_,*h*_2_] = [33,16]. The state feature sets extracted from single fault data by VMD-RCMFE are input into the SSA-BP model, and the identification results of single faults are shown in Figure 18.

As can be seen from Figure 18, the proposed method achieves desirable fault recognition performance with all faults accurately identified, which verifies the effectiveness of the proposed method in single fault feature extraction and fault diagnosis. To further verify the superiority of this method, the following two aspects are analyzed.

(1) To verify the classification effect of SSA-BP, BP, KNN and DT are used to classify the single fault state feature set composed of RCMFE values of COA-VMD-reconstructed signals at specified scale factors. For the BP neural network, the network structure is 15-20-10-4, the maximum number of iterations is 500, the learning rate is 0.01, and the minimum training target error is 1 × 10^−4^. For the KNN classifier, a grid search strategy is adopted to traverse different *k* values in the set {3, 5, 7, 9, 11, 13}, and three-fold cross-validation is used to evaluate the model performance corresponding to each *k* value. The DT classifier selects entropy as the splitting criterion.

Multiple evaluation indicators, including accuracy (Acc), precision (Pre), recall (Rec), F1-score (F1) [40] and computational time (CT), are established, and the obtained results (the mean ± standard deviation of 10 runs) are shown in Table 5.

It can be seen from Table 5 that in the single fault classification task, all four methods attain a mean test accuracy above 97%, indicating good overall performance. The SSA-BP classifier achieves the highest mean accuracy of 99.38% (best run 100%), followed by DT at 98.75%, standard BP at 98.33% and KNN at 97.50%. The main reasons for these results are as follows: SSA determines the optimal hidden layer structure through global optimization, effectively avoiding the local optimum problem of standard BP gradient descent; DT quickly constructs clear axis-parallel decision boundaries for the highly separable single fault features; KNN is weaker owing to distance distortion in the high-dimensional feature space and its sensitivity to noise; and standard BP has a small number of misclassifications due to its initial weights and local optima.

In terms of computational time, SSA-BP requires the longest time of 186.42 s because the outer SSA performs about 500 BP trainings (a population of 50 over 10 iterations) to search for the optimal structure; this cost, however, is incurred only once in the offline stage. KNN takes 2.77 s, slightly longer than standard BP (1.83 s), because its grid search over six k values with three-fold cross-validation involves repeated predictions, whereas BP is trained only once. DT is the most time-efficient at only 0.41 s. These results indicate that the superior accuracy of SSA-BP is obtained at the expense of a higher offline computational cost, while its online prediction remains fast.

(2) To verify the effectiveness of COA-VMD in signal processing, EMD, EEMD, CEEMDAN and VMD with default parameters are used to decompose the same gear single fault vibration signal. According to the steps in this paper, the above methods are used to reconstruct the gear signals, and the RCMFE values of the reconstructed signals at specified scale factors are extracted to construct fault feature sample sets, which are input into SSA-BP for fault classification. The obtained results are shown in Table 6. The default parameter VMD method adopts the general setting, with *K* = 4 and *α* = 2000.

It can be seen from Table 6 that the COA-VMD method achieves the highest mean test accuracy of 99.38% (best run 100%), significantly outperforming the other four decomposition methods. EEMD ranks second at about 98.33%, default parameter VMD ranks third at about 96.67%, and CEEMDAN and EMD perform relatively poorly at 94.17% and 92.50%, respectively. The main reasons for these results are: COA-VMD adaptively optimizes K and α according to the signal characteristics, accurately decomposes the signal and suppresses mode aliasing; EMD has inherent endpoint effect and mode aliasing problems that cause loss of fault information; EEMD alleviates mode aliasing by adding white noise and averaging but still leaves noise residue; CEEMDAN further reduces the noise problem but cannot fundamentally overcome the defects of recursive decomposition; and default parameter VMD uses fixed *K* = 4 and *α* = 2000, which cannot adapt to all signals and is prone to under- or over-decomposition.

Regarding computational time, COA-VMD takes the longest time of 277.43 s, as the COA optimization requires about 500 VMD runs before the optimal *K* and *α* are determined; this optimization is performed once offline for each fault category. CEEMDAN (46.27 s) and EEMD (32.09 s) are also relatively time-consuming because they require hundreds of noise-assisted EMD realizations, with CEEMDAN being more expensive owing to its additional adaptive noise operations. In contrast, the single-run EMD (4.35 s) and default VMD (4.22 s) are much faster, and their time is dominated by the common RCMFE feature extraction. Thus, the adaptive parameter optimization of COA-VMD substantially increases the offline computational burden, which is the price of its superior decomposition and diagnostic performance.

### 5.2. Compound Fault Diagnosis

**Figure 19:**
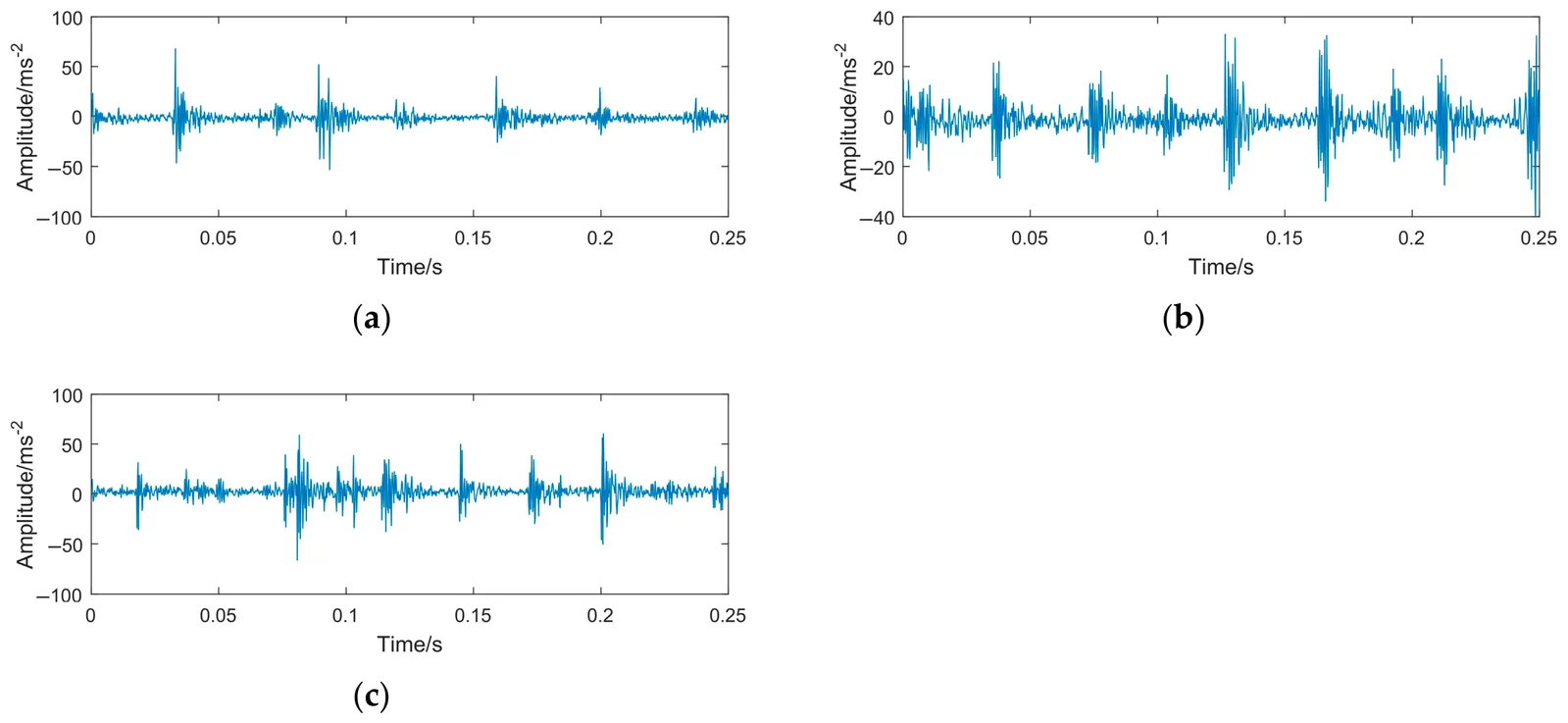
Time-domain waveform of the compound fault signal. (**a**) Normal state; (**b**) tooth breakage-wear; (**c**) pitting-wear.

To further verify the feature separability and stable within-distribution recognition of the proposed method, the data with labels 5, 6 and 7 are used for the experiment. A group of data is randomly selected, and the time-domain waveforms are shown in Figure 19.

Similar to the single fault signals, the collected compound fault signals also contain a large amount of noise interference components. Therefore, it is necessary to denoise the signals to improve the accuracy of feature extraction. The COA is used to optimize the key parameters of VMD, and the parameter settings of the COA are the same as those in the single fault experiment. The optimization results are shown in Table 7.

The same label-free logic applies to the compound fault data: the values in Table 7 result from the unsupervised CI optimization on each signal and are reported for illustration; in deployment, the VMD parameters are fixed from the development stage, and the test signals are decomposed without using their fault categories.

For the compound fault signals, the coupling of breakage/pitting impacts with wear sidebands broadens the effective frequency bands: a relatively smaller *α* (*α* = 1343 for tooth breakage-wear) allows moderately wider bands to accommodate the coupled fault components, whereas pitting-wear, with weaker and more distributed impacts, requires a larger *α* (*α* = 2133); both compound faults involve more coupled oscillation modes than the normal state, giving *K* = 5. These results again confirm the necessity of adapting *K* and *α* to each fault signal rather than using fixed parameters.

**Figure 20:**
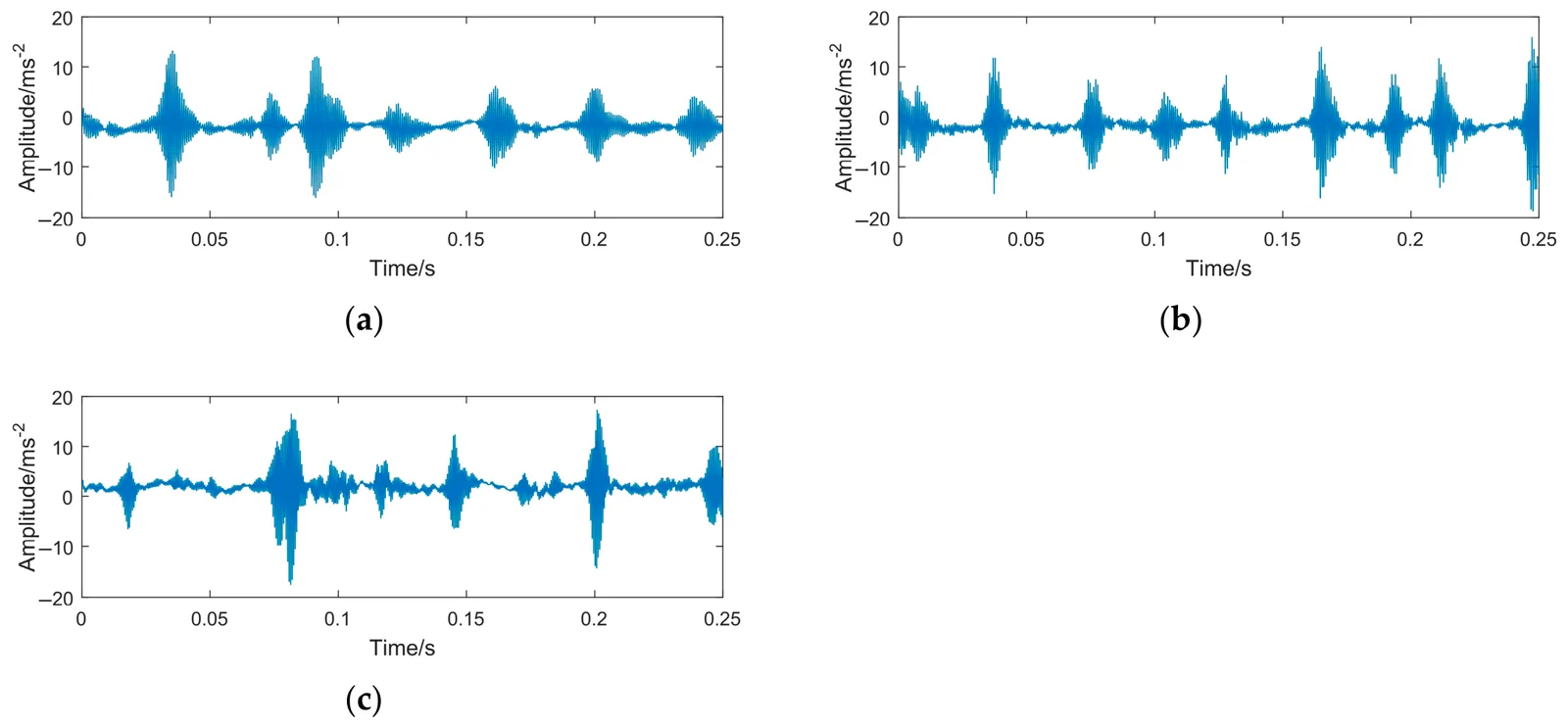
Time-domain waveform of the compound fault reconstructed signal. (**a**) Normal state; (**b**) tooth breakage-wear; (**c**) pitting-wear.

According to these results, a randomly selected group of compound fault signals is decomposed, and the CEF values are calculated. Sensitive IMF components are selected for signal reconstruction, and the time-domain waveforms of the reconstructed signals are shown in Figure 20.

It can be seen from Figure 20 that the noise interference components in the compound fault reconstructed signals are effectively filtered out, and the impact components in the signals are highlighted, which guarantees subsequent signal feature extraction.

**Figure 21:**
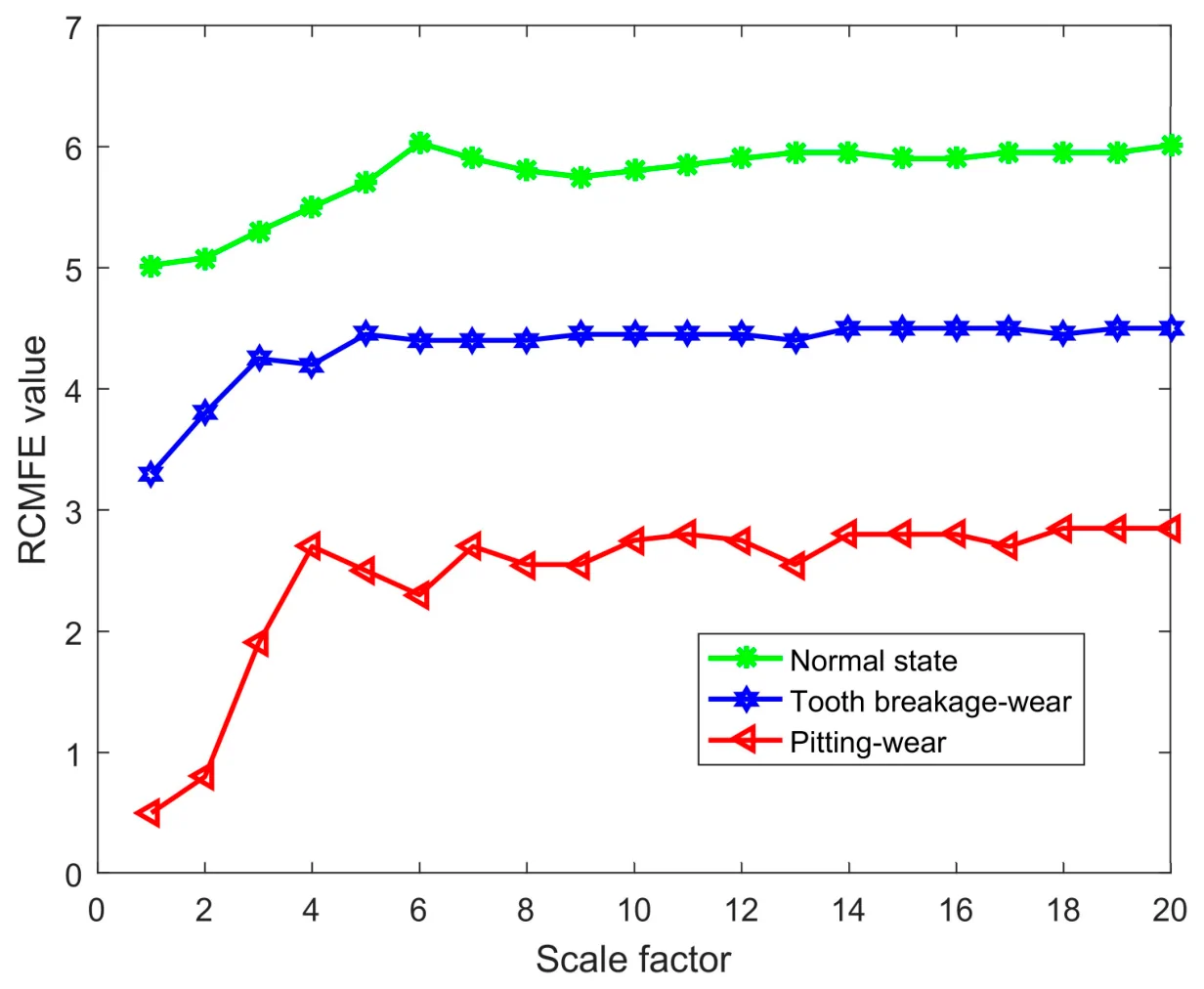
RCMFE values of compound fault reconstructed signals.

The RCMFE curves of each reconstructed signal are calculated, and the results are shown in Figure 21. The parameter settings of RCMFE are the same as those in the single fault experiment.

It can be seen from Figure 21 that the RCMFE values of the three different gear vibration signals generally show an upward trend as the scale factor increases. Moreover, the entropy value curves have good separability and strong stability, and no cross-overlapping phenomenon occurs, indicating good performance in signal fault extraction and fault discrimination.

**Figure 22:**
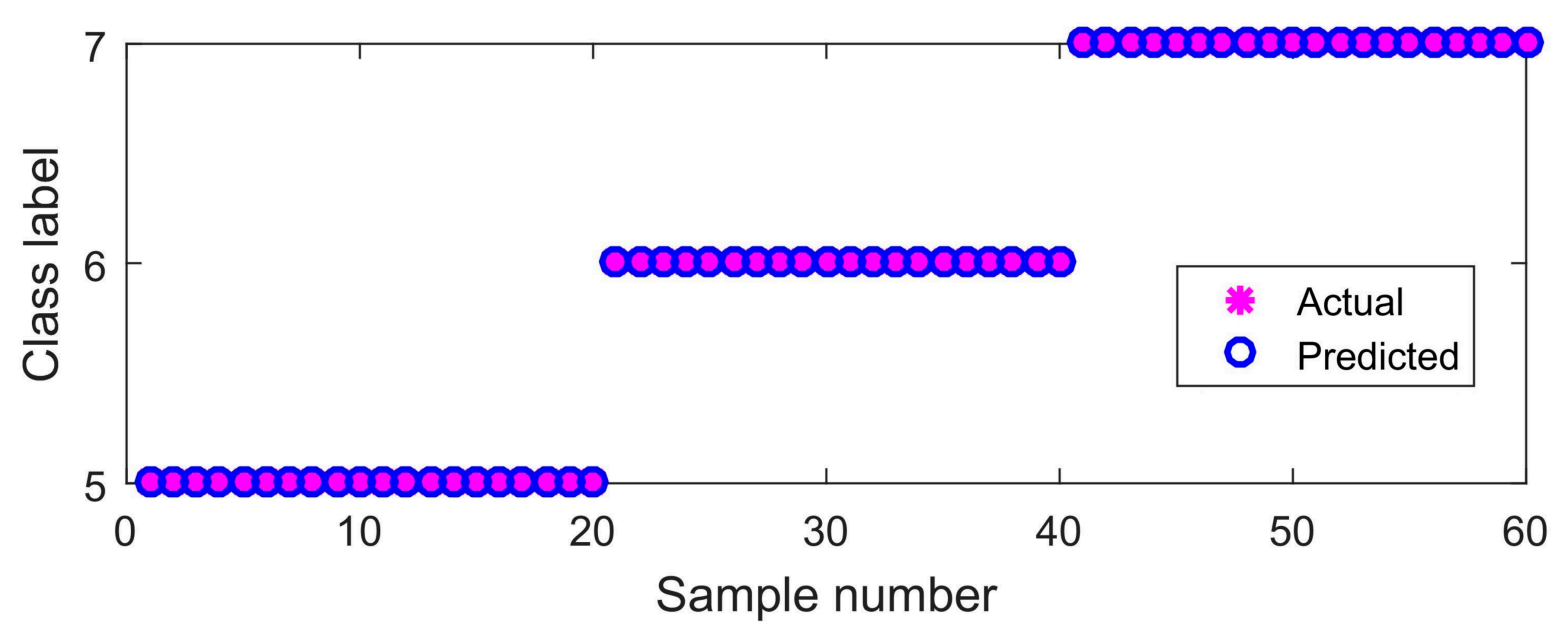
The identification results of compound faults.

To achieve accurate classification of fault types, the SSA is used to optimize the number of neurons in the hidden layers of BP. The parameter settings of the SSA are the same as those in the single fault experiment, and the optimization result is [*h*_1_, *h*_2_] = [30, 48]. The identification results of compound faults are shown in Figure 22. As can be seen in Figure 22, the proposed method also achieves desirable identification performance in the more complex compound fault classification task, with all compound faults accurately recognized.

To further verify the superiority of the proposed method in terms of feature separability and stable within-distribution recognition, the following two aspects are analyzed.

(1) To verify the classification effect of SSA-BP, BP, KNN and DT are used to classify the compound fault state feature set composed of RCMFE values of COA-VMD-reconstructed signals at specified scale factors. The parameter settings of KNN and DT are the same as those in the single fault experiment, the BP neural network structure is 15-14-6-3, and the remaining parameters are also the same as those in the single fault experiment. The diagnostic results of different classification methods are shown in Table 8.

It can be seen from Table 8 that, in the more complex compound fault task, the SSA-BP classifier still achieves the highest mean accuracy of 98.33% (best run 100%), outperforming the other three classifiers; standard BP follows at about 96.67%, while DT and KNN are around 95.83% and 95.00%. Compared with the single fault results, all classifiers degrade to different degrees. The main reasons for these results are: the mutual coupling and interference of compound fault features reduce the inter-class differences and increase the classification difficulty; SSA-BP fits complex nonlinear mappings and balances learning and generalization through the optimized structure, so it degrades the least; DT can only build axis-parallel boundaries and cannot handle complex nonlinear relationships, so it declines clearly; and KNN suffers from high-dimensional distance distortion, while standard BP is prone to local optima, both showing additional misclassifications.

The computational time shows a pattern similar to that of the single fault case: SSA-BP still requires the longest time (158.73 s) because of repeated BP training in the SSA search, while BP, KNN and DT only take 1.45 s, 2.18 s and 0.25 s, respectively. All the values are slightly smaller than those in Table 5 because the compound fault feature set contains fewer samples. This confirms that the SSA optimization is the dominant computational cost of the classification stage, which can be completed offline, whereas the trained SSA-BP responds almost instantaneously.

(2) To verify the effectiveness of COA-VMD feature extraction, EMD, EEMD, CEEMDAN and VMD with default parameters are used to decompose the same gear compound fault vibration signal. According to the steps in this paper, the above methods are used to decompose and reconstruct the gear signals, and feature extraction is performed on the reconstructed signals to construct fault feature sample sets, which are input into SSA-BP for fault classification. The obtained results are shown in Table 9. The default parameter VMD method adopts the general setting, with *K* = 4 and *α* = 2000.

It can be seen from Table 9 that, in the compound fault task, the COA-VMD method still maintains the highest mean accuracy of 98.33% (best run 100%), whereas the other four decomposition methods degrade markedly. Default parameter VMD ranks second at about 81.67%; EMD, CEEMDAN and EEMD all fall below 70%, with EEMD ranking the lowest at about 60.83%. Compared with the single fault results, all comparative decomposition methods decline significantly. The main reasons for these results are: COA-VMD adaptively adjusts the decomposition parameters, separates coupled multi-fault features into different IMFs and enhances weak fault information through the CEF; EMD, EEMD and CEEMDAN suffer from severely aggravated mode aliasing on compound faults and cannot effectively separate different fault features, so their accuracies fall below 70%; and default parameter VMD cannot adapt to the complex frequency characteristics with fixed parameters, which aggravates mode aliasing and over-decomposition and disperses or masks some fault features.

The computational times in Table 9 are much shorter than those in Table 6 because the compound fault signals contain only 1280 points. Nevertheless, COA-VMD still takes the longest time (18.36 s), as the COA optimization repeats hundreds of VMD runs, whereas EMD, EEMD, CEEMDAN and default VMD only take 0.28 s, 1.14 s, 1.63 s and 0.29 s, respectively. The large gap between COA-VMD and the other methods again demonstrates that adaptive parameter optimization is the main computational cost, while the single-sample online processing of all methods is fast for such short signals.

### 5.3. Verification of the Rationality of CEF Weights

**Figure 23:**
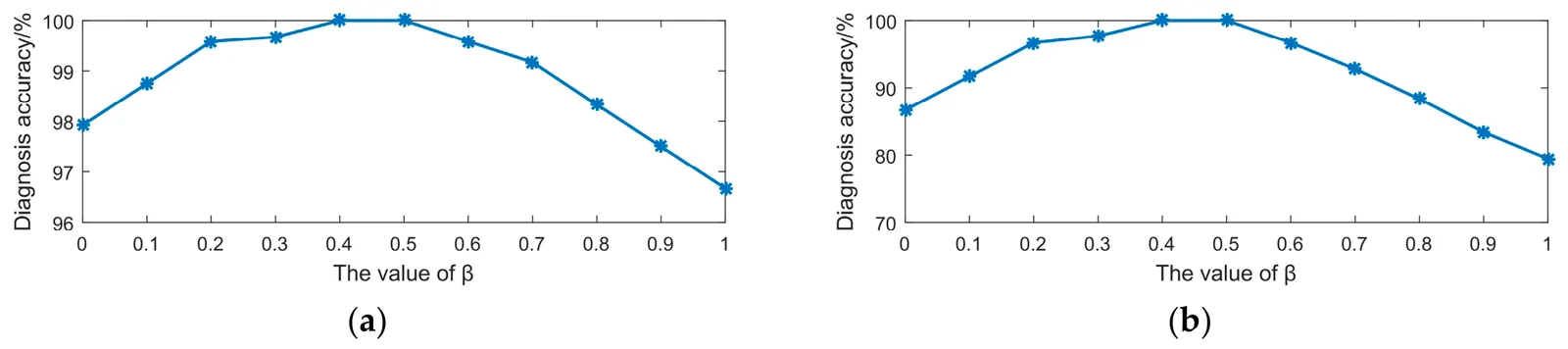
Diagnosis accuracy corresponding to different values of *β*. (**a**) Signal fault datasets; (**b**) compound fault datasets.

To verify the rationality of the weights *β* and *γ* in Equation (9) and to examine whether they remain applicable across different fault signals, a weight sensitivity analysis is conducted on both the single and compound fault datasets. Setting *γ* = 1 − *β* and varying *β* from 0 to 1.0 in steps of 0.1, the complete CEF-based IMF screening, signal reconstruction, RCMFE feature extraction and SSA-BP classification procedure is repeated for each weight, and the average diagnostic accuracy over 10 independent runs is reported in Figure 23.

The results show that the accuracy varies with *β* in a consistent inverted-U pattern on both datasets. When *β* = 0.4 and *β* = 0.5, the single and compound fault datasets both reach the highest diagnostic accuracy; as *β* moves away from this range toward either endpoint, the accuracy decreases monotonically, and the decline is more pronounced for the compound faults, whose coupled features are more sensitive to the component screening criterion. Specifically, relying solely on the frequency-domain cross-correlation (*β* = 0) gives accuracies of 97.92% and 86.67%, whereas relying solely on the energy entropy increment (*β* = 1) gives even lower accuracies of 96.67% and 79.44%, confirming that frequency-domain information is relatively more critical and that either single criterion is inferior to their fusion. The fact that two independent datasets, with different signal lengths, test benches and fault configurations, attain a common optimum at *β* = 0.4–0.5 demonstrates that the chosen *β* = 0.4, *γ* = 0.6 is optimal rather than empirically assumed and remains valid across different fault signals. This validates both the optimality and cross-fault applicability of the CEF weights.

### 5.4. Unbiased Analysis of Hyperparameters

To avoid subjective (biased) hyperparameter settings, all hyperparameters used in this work are summarized in Table 10 together with their determination basis. The VMD parameters and the BP structure are determined automatically by the COA and SSA optimizers rather than manual tuning, the CEF weights are verified through the sensitivity analysis in Section 5.3, and the remaining parameters follow the established literature or parameter trials; therefore, no critical hyperparameter is set by arbitrary personal judgment. The hyperparameter settings and their determination bases for unbiased analysis are shown in Table 10.

Finally, the difference from the authors’ earlier work [10] should be clarified. Reference [10] addresses signal denoising by combining VMD with MODWPT and does not perform fault classification; in contrast, the present work introduces the label-free CI for VMD parameter optimization, the CEF for automatic sensitive-IMF selection, RCMFE feature extraction and an SSA-optimized BP classifier, forming a complete diagnosis chain, in which the CI and the CEF are the new mathematical components. In addition, the present held-out tests are conducted under essentially the same experimental condition and therefore assess within-distribution discrimination rather than generalization across different working conditions; cross-condition and cross-component generalization, together with end-to-end deep learning, are left as future work.

## 6. Conclusions

Aiming at the difficulties in extracting gear fault feature information and the unsatisfactory performance of commonly used machine learning classification models in terms of feature separability and stable within-distribution recognition, this paper proposes a fault diagnosis method that combines COA-VMD, RCMFE and SSA-BP. This method organically combines the effectiveness of COA-VMD in signal decomposition, the superiority of RCMFE in feature extraction and the accuracy of SSA-BP in classification and recognition. Its effectiveness is verified through measured data of gear single and compound faults, and a comprehensive comparison with various commonly used methods is conducted. The main conclusions are as follows:

(1) A complete gear fault signal processing and feature extraction scheme is proposed. Firstly, the COA is used to optimize the key decomposition parameters of VMD, effectively solving the blindness of traditional manual parameter tuning. Secondly, a sensitive IMF component selection method based on a time–frequency-domain comprehensive evaluation factor is proposed, which comprehensively considers time-domain energy characteristics and frequency-domain cross-correlation characteristics, automatically eliminates noise and background signal interference, and avoids the subjectivity of manual threshold setting. Finally, RCMFE is used to extract fault features; compared with MFE and CMFE, RCMFE completely retains the time-domain detail information at different scales, and its entropy value curves have better stability and separability, which can accurately characterize the subtle differences in signal nonlinear complexity under different gear fault states. In addition, a weight sensitivity analysis conducted on both the single- and compound fault datasets shows that CEF weights β = 0.4 and γ = 0.6 produce the highest diagnostic accuracy and remain valid across different fault signals, confirming the rationality and general applicability of the CEF.
(2) A BP neural network classification model optimized by the SSA is constructed. Using the strong global optimization capability of the SSA, the number of neurons in the two hidden layers is automatically determined, which avoids the over-fitting or under-fitting caused by manual network setting and improves the classification accuracy and stability. Under the unbiased train/validation/test protocol, this model achieves the highest mean test accuracy of 99.38% for single faults (best run 100%), while in the compound fault task with severe feature coupling, its advantage is most evident, again attaining the highest mean accuracy of 98.33% (best run 100%) and clearly outperforming standard BP, KNN and DT.
(3) The proposed method performs well in both gear single fault and compound fault diagnosis. On the independent, previously unseen test sets, it reaches mean accuracies of 99.38% and 98.33% (best run 100% for both) for the single- and compound fault tasks. Compared with EMD, EEMD, CEEMDAN and default parameter VMD, the COA-VMD method gives higher compound fault accuracies by 31.11, 37.50, 34.16 and 16.66 percentage points, respectively, demonstrating its superiority for strongly nonlinear and strongly coupled signals.
(4) The computational efficiency of the proposed method was evaluated through both theoretical complexity analysis and measured computational time. COA-based VMD optimization and SSA-based BP training are the most time-consuming stages, but they are performed only once offline; the online stage is dominated by the RCMFE calculation, whose complexity is approximately quadratic in sequence length, whereas single-sample prediction is nearly instantaneous. These results indicate that high diagnostic accuracy is obtained at the cost of an increased offline computational burden and that the current method is more suitable for offline analysis or periodic inspection than for strictly real-time online monitoring.

The method proposed in this paper provides a new and effective approach to gear fault diagnosis, but there are still some limitations that need further research. Firstly, this paper only verifies the effectiveness of the method on gear data from two different test benches; in the future, verification will be conducted on single and compound fault data collected simultaneously on the same test bench, and the method will be extended to more fault types and damage degrees. Secondly, the applicability of the method to other rotating mechanical components such as rolling bearings and rotors will be studied to verify its broader generalization ability. Thirdly, the current framework is computationally demanding for online deployment: because RCMFE repeatedly computes fuzzy entropy over 20 scales with approximately quadratic complexity in sequence length, the online feature extraction stage may not satisfy the real-time requirements of high-speed equipment, whereas the COA and SSA optimization stages can be completed offline; scale subset selection, incremental sliding-window entropy computation and parallel/GPU acceleration will therefore be investigated to improve the real-time performance. Finally, the combination of the proposed method with deep learning technology will be explored to realize end-to-end intelligent fault diagnosis and further improve diagnostic efficiency and automation levels.

## Figures and Tables

**Table 1:** Single fault status.

State	Label
Normal state	1
Crack occurs in the root	2
Worn gear	3
Gear with a broken tooth	4

**Table 2:** Compound fault status.

State	Label
Normal state	5
Tooth breakage-wear	6
Pitting-wear	7

**Table 3:** Optimization results of different algorithms for VMD parameters.

Model	[*K*,*α*]	Best Fitness Value	Convergence Iteration	Mean ± Std
COA-VMD	[4,3736]	0.95544	4	0.9557 ± 0.0002
GWO-VMD	[5,3314]	0.96304	8	0.9639 ± 0.0009
WOA-VMD	[4,3428]	0.96113	7	0.9620 ± 0.0008
PSO-VMD	[5,3086]	0.96418	6	0.9652 ± 0.0011
MFO-VMD	[4,3512]	0.96573	5	0.9668 ± 0.0014
GA-VMD	[6,2647]	0.96856	9	0.9701 ± 0.0018

**Table 4:** Optimal parameters for a single fault COA-VMD.

State	[*K*,*α*]
Normal state	[6,1716]
Crack occurs in the root	[4,3736]
Wear gear	[4,2221]
Gear with a broken tooth	[6,2605]

**Table 5:** Comparison of different classifiers for single fault diagnosis.

EI	Classifier			
	SSA-BP	BP	KNN	DT
Acc	99.38 ± 0.83%	98.33 ± 1.27%	97.50 ± 1.46%	98.75 ± 1.05%
Pre	99.40 ± 0.85%	98.38 ± 1.30%	97.55 ± 1.48%	98.78 ± 1.08%
Rec	99.36 ± 0.88%	98.30 ± 1.33%	97.92 ± 1.40%	98.72 ± 1.10%
F1	99.37 ± 0.86%	98.33 ± 1.31%	97.66 ± 1.45%	98.75 ± 1.09%
CT	186.42 s	1.83 s	2.77 s	0.41 s

**Table 6:** Comparison of different signal decomposition methods for single fault diagnosis.

EI	Signal Decomposition Methods				
	COA-VMD	EMD	EEMD	CEEMDAN	VMD
Acc	99.38 ± 0.83%	92.50 ± 1.83%	98.33 ± 1.27%	94.17 ± 1.69%	96.67 ± 1.45%
Pre	99.40 ± 0.85%	92.30 ± 1.86%	98.31 ± 1.30%	94.42 ± 1.72%	96.64 ± 1.48%
Rec	99.36 ± 0.88%	93.48 ± 1.78%	98.36 ± 1.33%	94.52 ± 1.70%	96.70 ± 1.46%
F1	99.37 ± 0.86%	92.33 ± 1.84%	98.33 ± 1.31%	94.23 ± 1.71%	96.66 ± 1.47%
CT	277.43 s	4.35 s	32.09 s	46.27 s	4.22 s

**Table 7:** Optimal COA-VMD parameters for compound fault signals.

State	[*K*,*α*]
Normal state	[6,2889]
Tooth breakage-wear	[5,1343]
Pitting-wear	[5,2133]

**Table 8:** Comparison of different classifiers for compound fault diagnosis.

EI	Classifier			
	SSA-BP	BP	KNN	DT
Acc	98.33 ± 1.61%	96.67 ± 2.18%	95.00 ± 2.45%	95.83 ± 2.28%
Pre	98.40 ± 1.64%	96.75 ± 2.20%	95.15 ± 2.48%	95.55 ± 2.31%
Rec	98.27 ± 1.67%	96.62 ± 2.23%	95.22 ± 2.42%	96.05 ± 2.26%
F1	98.33 ± 1.65%	96.67 ± 2.21%	95.14 ± 2.46%	95.74 ± 2.29%
CT	158.73 s	1.45 s	2.18 s	0.25 s

**Table 9:** Comparison of different signal decomposition methods for compound fault diagnosis.

EI	Signal Decomposition Methods				
	COA-VMD	EMD	EEMD	CEEMDAN	VMD
Acc	98.33 ± 1.61%	67.22 ± 3.15%	60.83 ± 3.42%	64.17 ± 3.28%	81.67 ± 2.63%
Pre	98.40 ± 1.64%	63.70 ± 3.30%	64.30 ± 3.55%	67.12 ± 3.36%	81.45 ± 2.68%
Rec	98.27 ± 1.67%	61.78 ± 3.42%	60.70 ± 3.60%	65.50 ± 3.30%	81.80 ± 2.60%
F1	98.33 ± 1.65%	61.20 ± 3.38%	60.45 ± 3.50%	63.60 ± 3.34%	81.62 ± 2.64%
CT	18.36 s	0.28 s	1.14 s	1.63 s	0.29 s

**Table 10:** Hyperparameter settings and their determination bases for unbiased analysis.

Module	Hyperparameter	Value	Determination Basis
COA	Population size/iterations	50/10	References [20,34]
VMD search range	*K*/α	[2,7]/[100,5000]	References [24,27]
CEF	Weights *β*/*γ*	0.4/0.6	Section 5.3
RCMFE	*m*/*n*/*r*/*τ*_max_	2/2/0.2 SD/20	References [35,36], parameter trials
SSA	Population size/iterations	50/10	References [20,23]
BP	Hidden layers/max epochs/learning rate/target error	2/500/0.01/1 × 10^−4^	References [38,39]

## Data Availability

The testing datasets that support the conclusions of this article will be made available by the authors upon request.

## Abbreviations

The following abbreviations are used in this manuscript:

VMD	Variational Mode Decomposition
COA	Coati Optimization Algorithm
SSA	Sparrow Search Algorithm
IMF	Intrinsic Mode Function
RCMFE	Refined Composite Multiscale Fuzzy Entropy
EMD	Empirical Mode Decomposition
EEMD	Ensemble Empirical Mode Decomposition
GWO	Grey Wolf Optimization
WOA	Whale Optimization Algorithm
PE	Permutation Entropy
SE	Sample Entropy
DE	Dispersion Entropy
MSE	Multiscale Entropy
KNN	K-Nearest Neighbor
DT	Decision Tree
BP	Back-Propagation
CI	Composite Index
CEF	Comprehensive Evaluation Factor
MIE	Mutual Information Entropy
MFE	Multiscale Fuzzy Entropy
CEEMDAN	Complete Ensemble Empirical Mode Decomposition with Adaptive Noise
PSO	Particle Swarm Optimization
MFO	Moth–Flame Optimization
GA	Genetic Algorithm
CMFE	Composite Multiscale Fuzzy Entropy
Acc	Accuracy
Pre	Precision
Rec	Recall
F1	F1-Score
CT	Computational Time
